# Fe–Mn nanocomposites doped graphene quantum dots alleviate salt stress of *Triticum aestivum* through osmolyte accumulation and antioxidant defense

**DOI:** 10.1038/s41598-023-38268-6

**Published:** 2023-07-07

**Authors:** Md Salman Haydar, Salim Ali, Palash Mandal, Debadrita Roy, Mahendra Nath Roy, Sourav Kundu, Sudipta Kundu, Chandrani Choudhuri

**Affiliations:** 1 Nanobiology and Phytotherapy Laboratory, Department of Botany, University of North Bengal, Siliguri, West Bengal 734013 India; 2 Department of Chemistry, University of North Bengal, Darjeeling, West Bengal 734013 India; 3 Department of Chemistry, Alipurduar University, Alipurduar, West Bengal 734013 India; 4 Department of Botany, North Bengal St. Xavier’s College, University of North Bengal, Rajganj, Jalpaiguri, West Bengal 735134 India

**Keywords:** Biochemistry, Physiology, Plant sciences, Nanoscience and technology

## Abstract

An investigation was carried out to evaluate the effect of graphene quantum dots (GQD) and its nanocomposites on germination, growth, biochemical, histological, and major ROS detoxifying antioxidant enzyme activities involved in salinity stress tolerance of wheat. Seedlings were grown on nutrient-free sand and treatment solutions were applied through solid matrix priming and by foliar spray. Control seedlings under salinity stress exhibited a reduction in photosynthetic pigment, sugar content, growth, increased electrolyte leakage, and lipid peroxidation, whereas iron-manganese nanocomposites doped GQD (FM_GQD) treated seedlings were well adapted and performed better compared to control. Enzymatic antioxidants like catalase, peroxidase, glutathione reductase and NADPH oxidase were noted to increase by 40.5, 103.2, 130.19, and 141.23% respectively by application of FM_GQD. Histological evidence confirmed a lower extent of lipid peroxidation and safeguarding the plasma membrane integrity through osmolyte accumulation and redox homeostasis. All of these interactive phenomena lead to an increment in wheat seedling growth by 28.06% through FM_GQD application. These findings highlight that micronutrient like iron, manganese doped GQD can be a promising nano-fertilizer for plant growth and this article will serve as a reference as it is the very first report regarding the ameliorative role of GQD in salt stress mitigation.

## Introduction

In the course of their life, plants face a multitude of environmental anomaly. A series of defensive mechanisms including biochemical, molecular, and physiological processes play synchronous roles to counteract and adapt to the concerned abiotic and biotic stress^1^. Among major cereals, wheat (*Triticum aestivum*) gains an essential position for ensuring food and nutritional security. In South-East Asian countries like India, Pakistan, Nepal, and Bangladesh, wheat serves as the second major staple crop after rice. However rapidly increasing soil and water salinity creates a severe threat to the productivity of wheat globally^2^. It is projected that 20% of the global cultivable land is under threat of salt stress^3^. While in India the area is around 6.727 million hectares, which is approximately 2.1% of the total geographical area of the country^4^. Among various field crops, wheat is generally more susceptible to salinity that negatively affects the growth and development of wheat plants leading to diminished grain yield and quality or even complete crop failure under extreme saline conditions^2^.

Nanomaterials are believed to be the potential to combat the arisen challenges in the agricultural sector. In recent decades, a wide number of nanomaterials have been found for taking consideration to improve crop productivity, combat diseases and pests, enhance the efficacy of fertilizer and pesticides, monitor crop health, and most importantly tomanage environmental stresses^5,6^. Various studies also revealed the defensive role of nanoparticles in the mitigation of biotic stress, especially in the alleviation of salt stress^7–9^. As a member of the carbon nanomaterials category, graphene quantum dots (which are small pieces of two-dimensional graphene of a size range below 100 nm) became a rising star in this class due to their appreciable biocompatibility and features like optical and fluorescence properties and inherent photoluminescence capability^10^. In addition to that, carbon-based nanoparticles have been reported to alleviate the adverse effects caused by salinity and associated abiotic stresses^11^. Some recent research reported that GQD has comparatively less or no toxicity on biological materials, has adequate biocompatibility, and easy functionalization with other biomolecules and chemical entities^12–15^. Furthermore, the high hydrophilicity and appreciable cell permeability of this kind of material make them appropriate for water-based applications in biological system^16,17^. GQDs have single atomic layer plane conjugate structure, large surface area, and oxygen-containing group which offer active binding site to load and/or carry the drug and other kinds of molecules^10^. Doping of GQDs with specific molecules, hetero-atoms, nanomaterials, DNA strands, and enzymes has already been reported^18^. Hetero-atoms doped inside carbon-based nanomaterials like GQDs can effectively regulate their fundamental properties including surface and local chemical characteristics^19,20^. For example, graphene doped with nitrogen could efficiently modulate the band gap of the host molecule (GQDs) to introduce new properties^21^. A wide number of researchers also reported the ability of nanoparticles like silver, titanium oxide, zinc oxide, and carbon nano-tubes in the germination and seedling growth of wheat^22,23^. Concerning this, we have hypothesized that GQD in combination with essential micronutrients like iron and manganese will act as an effective elicitor in the alleviation of plant growth by mitigating adverse effects generated through salt stress.

The current study deals with the synthesis of Fe–Mn nanocomposites doped graphene quantum dots (GQDs) using *Azadirachta indica* (neem) extract as a reducing agent and starch as a carbon source. The efficacy of this prepared nano-conjugate on germination, salinity stress mitigation (NaCl mediated), and seedling growth of bread wheat was compared with the efficiency of simple iron nanoparticles, iron-manganese (Fe–Mn) nanocomposites and normal GQDs. Moreover, synthesized nano-conjugates were applied through solid matrix priming (SMP), the process itself was reported for alleviation of salt stress^24,25^. After the application of different nanomaterials, germination-related parameters, stress-related index, phenotypic appearance of seedling, biochemical, enzymatic, and non-enzymatic antioxidant and oxidative stress (both qualitative and quantitative) attributes were examined to evaluate the effectiveness of the applied formulations. Furthermore, to the best of our knowledge, this is probably the first report to explore the role of GQD and its nano-conjugate on salinity stress mitigation as zero-dimensional carbon-based nano-materials like GQDs as plant growth regulator has not been explored widely.

## Material and methods

### Synthesis of graphene quantum dots (GQDs)

With small modifications, the method described by Chen et al.^26^ was followed for the preparation of graphene quantum dots (GQD) using starch (easily available natural carbohydrate) as a carbon source. To stay away from any kind of acids or bases, only starch and hydrogen peroxide are used as a precursor. For that, 1 g of starch was dissolved in 20 mL of deionised water under continuous stirring maintaining a steady temperature (60 °C) until a clear solution was formed. Meanwhile, two drops of H_2_O_2_ (30%) was added and the entire mixture solution was transferred into a stainless steel Teflon-lined autoclave and kept for 3 h at 160 °C. The solution was then allowed to cool to room temperature and after centrifugation at 8000 rpm, a yellow-colored solution was obtained which was then stored for further use.

### Preparation of *Azadirachta indica* leaf extracts

Fresh neem (*Azadirachta indica)* leaves of different ages (mid-aged and mature) were collected from the University of North Bengal campus (26° 42′ 35″ N and 88° 21′ 05″ E) and washed with distilled water to remove any impurities if present on the leaf surface. This plant naturally grows in large numbers throughout India and is not included under RET (rare, endangered, threatened) category of IUCN. However, for collection, we took the permission from institutional ethical committee, Alipurduar University, India (Ref. No.: APDU/IEC/2021/11, dated: 27/10/2021). The collected plant sample was identified by Dr. Monoranjan Chowdhury, herbarium in-charge and professor of plant taxonomy and biosystematics, Department of Botany, University of North Bengal and for future reference voucher specimen has been deposited in NBU herbarium (Voucher No. NBU/2022/112). The plant study complies with relevant institutional, national, and international guidelines and legislation.

Clean leaves (a mixture of mid-aged and mature neem leaves in a ratio of 1:1) were then chopped into small pieces using scissors and dried in a hot air oven at 60 °C for 72 h. Thereafter, dried leaves (10 g) were heated (at 80 °C) in deionized (DI) water (100 mL) for one hour under continuous stirring. Subsequently, the homogeneous solution obtained after filtration was cooled to room temperature and finally stored at 4 °C for future use.

### Synthesis of iron nanoparticles (F-NP)

Iron nanoparticles were synthesized by the facile one-step green method using neem leaf extract as a reducing agent. For synthesis, a homogeneous solution of ferrous sulphate (2 g in 100 mL DI water) was prepared, and to that 10 mL of ethylene glycol was added which turn the colour of the solution into the green. Subsequently, 10 mL of neem extract was added drop-wise and left for another 30 min under continuous stirring. The development of a dark reddish brown colour solution indicated the formation of desired nanoparticles. Finally, the sample was dried by heating mantle.

### Synthesis of Fe–Mn nanocomposites (FM-NC)

Iron-manganese (Fe–Mn) nanocomposites were synthesized following green technology using neem leaf extract as a reducing and ethylene glycol as stabilizing agent. For that, ferrous sulphate (2 g) and manganese chloride (2 g) was dissolved in a beaker containing 150 mL of water and mixed well using a magnetic stirrer. Ethylene glycol (10 mL) was then added to the mixture and continued stirring for another 30 min. Subsequently, prepared neem extract was added drop by drop until the solution turns into reddish brown, indicating the formation of desired nanocomposites. Finally, the prepared solution was dried in a hot air oven (at 60 °C) to obtain nanocomposite powder.

### Synthesis of Fe–Mn nanocomposites doped graphene quantum dots (FM-GQD)

Fe–Mn nanocomposites were synthesized by following the same protocol as GQD. A homogeneous solution of starch (1 g) was prepared as previously and was labeled as ‘solution A’. Whereas ‘solution B’ was prepared separately by mixing manganese chloride (0.5 g) and ferrous sulphate (0.5 g) solution under stirring, followed by the addition of freshly prepared neem extract (5 mL). Subsequently, two solutions i.e. ‘solution A’ and ‘solution B’ were mixed and transferred into stainless steel Teflon lined autoclave and kept there for 3 h maintaining 160 °C temperature. After that resultant mixture was cooled down to room temperature and after centrifugation at 8000 rpm obtained dark yellow solution was collected at kept at 4 °C for future use.

### Characterization of synthesized nanomaterials

Surface morphology and average grain sizes of synthesized nanoparticles were determined using a scanning electron microscope (Jeol JSM-IT100). To find out the elemental composition of the prepared nano-materials, Energy-dispersive X-ray spectroscopy (EDS) analysis was also performed (OXFORD Instruments). The crystalline structure of the synthesized nano-conjugates was analyzed using X-ray diffractometer (XPERT-PRO PW3071 XRD instrument) using 40 kV accelerating voltage and 30 mA emission current and Cu Kα (λ = 1.5418 Ǻ) as target material. Dynamic light scattering (DLS) technique was used to measure the average hydrodynamic size and was done using the ZETASIZER NANO ZS90 ZEN3690 instrument.

### Experiment on germination and seedling growth of wheat

The experiment was conducted under natural photoperiod using plastic tray at Mulberry Germplasm and Experimental Garden, Department of Botany, University of North Bengal during February–March, 2021. The ‘PBW-343’ wheat (*Triticum aestivum* L.) cultivar was selected as a model plant and the seeds of this particular variety were collected from National Seeds Corporation Limited, New Delhi, India (a Government of India undertaking company). A factorial combination of four different kinds of nano-material (treatments) in addition to the control group and two different concentrations (200 and 500 µg/mL) were applied in the present study. Treatments were normal graphene quantum dots (applied in 200 and 500 µg/mL, abbreviated as GQD_2 and GQD_5 respectively), Fe–Mn nanocomposites doped graphene quantum dots (abbreviated as FM_GQD_2 and FM_GQD_5), Fe–Mn nanocomposites (abbreviated as FM_NC_2 for 200 µg/mL and FM_NC_5 for 500 µg/mL), Normal iron nanoparticles (abbreviated as F_NP_2 and F_NP_5) and control (hydroprimed and unprimed, abbreviated as C_HP and C_UP respectively). Treatment solutions were applied initially to the seeds through the solid matrix priming (SMP) technique and later to the seedling by foliar spray. For that, surface sterilization of collected wheat seeds was done using 4% sodium hypochlorite (NaCIO) and then solid matrix priming was achieved by taking celite as a matrix (seeds:celite = 1:1). Treatment solutions (elicitors) at different concentrations (i.e. 200 and 500 µg/mL) were applied to the matrix by maintaining 30% matrix moisture and after mixing well with seeds, they were kept overnight inside airtight plastic zip bag. Control hydroprimed (C_HP) seeds were achieved by priming in celite but only distilled water was used as elicitor solution. After washing out the matrix with distilled water, seeds were transferred to petri-plates and allowed to germinate inside the seed germinator (REMI, Model-SG-6S-5/09) adjusted to 20 ± 2 °C. To calculate various germination-related parameters, the number of seeds that germinated on each petri-plate on each day was recorded up to 3rd day of germination.

Using the germination data following parameters were studied with the formula mentioned by Sen et al.^27^

$$\mathrm{Germination\, Index }(\mathrm{GI})=\sum \frac{{\mathrm{G}}_{\mathrm{t}}}{{\mathrm{T}}_{\mathrm{t}}}$$

where G_t_ is the number of seeds germinated on t*th* day and the T_t_ is the number of days up to t*th* day.

$$\mathrm{Germination\, Percentage}=\left(\frac{\mathrm{Number\, of\, seeds\, germinated}}{\mathrm{total\, number\, of\, seeds}}\right)\times 100$$

$$\mathrm{Mean\, Germination\, Time }\left(\mathrm{MGT}\right)=\frac{\mathrm{\Sigma dn}}{\mathrm{\Sigma n}}$$

where (n) is the number of seeds germinated on the day (d) and (d) is the number of days counted from the beginning of germination.

$$\mathrm{Coefficient\, of\, velocity\, of\, germination }(\mathrm{CVG}) = (\frac{\mathrm{\Sigma Ni}}{\mathrm{\Sigma NiTi}})\times 100$$

where N is the number of seeds germinated on day (i) and T is the number of daysfrom germination.

$${\text{Promptness}}\;{\text{Index}}\;\left( {{\text{PI}}} \right) = \left( {{\text{N}}_{{\text{t}}} {2} \times {1}} \right) + \left( {{\text{N}}_{{\text{t}}} {3} \times 0.{67}} \right) + ({\text{N}}_{{\text{t}}} {4} \times 0.{33})$$

where N is the number of seeds germinated at t*th* day.

### Assessment of seedling growth under NaCl-mediated salinity stress

Germinated seedlings were transplanted in sand-filled plastic trays (length-42.5 cm, breadth-30.5 cm, height-6.5 cm). Prior to use, the sand samples were acid washed using 0.05 M sulfuric acid solution to dislocate any cations (viz. Ca, K, Fe, Mg ions) if remain bound in the cation exchange sites of sand. Subsequently, the acid-washed sands were rinsed (three times) with double distilled water to eliminate sulfate and completely nutrient-free sand was used as growth media to ensure that the seedling would have been receiving nutrients only from the applied treatments source. The transplanted seedlings were watered every day up to the 10th days after transplantation (DAT) with an adequate amount of double distilled water. To induce salinity, 25 mM NaCl solution was applied from 11 to 14th DAT, and after primary acclimatization, 50 mM NaCl solution was applied from 15 to 20th DAT and their appearance was monitored up to 25 DAT. In one set of control group same rate of NaCl stress has been imposed (abbreviated as C_HP_S and C_UP_S). While, another set of the control group was grown using double distilled water to assess the growth of the wheat seedling in normal growth conditions (i.e. non saline; abbreviated as C_HP and C_UP). Treatment details are presented in Table 1. Foliar application of all the treatments solution (maintaining the same dose applied in seed priming) was done by spraying at the 10th, 15th, and 20th DAT. Growth parameters like shoot and root length were measured using a centimeter scale, and root and shoot biomass was determined using digital weight balance (Sartorius, QUINTIX224-10IN) after uprooting the seedling at 25 DAT.

**Table 1:** Different types of treatments and salinity dosages applied in the present study.

Priming status	Applied nanomaterials	Applied dosage	Abbreviation used	Amount of salt stress (NaCl) imposed
Nanoprimed	Graphene quantum dots	200 µg/mL	GQD_2S	NaCl @25 mM from 11 to 14th DAT and @50 mM from 15 to 20th DAT
	Graphene quantum dots	500 µg/mL	GQD_5S	NaCl @25 mM from 11 to 14th DAT and @50 mM from 15 to 20th DAT
	Fe–Mn nanocomposites doped graphene quantum dots	200 µg/mL	FM_GQD_2S	NaCl @25 mM from 11 to 14th DAT and @50 mM from 15 to 20th DAT
	Fe–Mn nanocomposites doped graphene quantum dots	500 µg/mL	FM_GQD_5S	NaCl @25 mM from 11 to 14th DAT and @50 mM from 15 to 20th DAT
	Fe–Mn nano-composites	200 µg/mL	FM_NC_2S	NaCl @25 mM from 11 to 14th DAT and @50 mM from 15 to 20th DAT
	Fe–Mn nano-composites	500 µg/mL	FM_NC_5S	NaCl @25 mM from 11 to 14th DAT and @50 mM from 15 to 20th DAT
	Iron nanoparticles	200 µg/mL	F_NP_2S	NaCl @25 mM from 11 to 14th DAT and @50 mM from 15 to 20th DAT
	Iron nanoparticles	500 µg/mL	F_NP_5S	NaCl @25 mM from 11 to 14th DAT and @50 mM from 15 to 20th DAT
Hydroprimed	Primed with distilled water	–	C_HP_S	NaCl @25 mM from 11 to 14th DAT and @50 mM from 15 to 20th DAT
	Primed with distilled water	–	C_HP	Without any salt stress
Unprimed	–	–	C_UP-S	NaCl @25 mM from 11 to 14th DAT and @50 mM from 15 to 20th DAT
	–	–	C_UP	Without any salt stress

*DAT* days after transplantation.

### Assessment of stress tolerance index

After measuring root and shoot length and biomass of treated wheat seedlings, stress indices like plant height stress tolerance index (PHSI), root length stress tolerance index (RLSI), shoot length stress tolerance index (SLSI) and dry matter stress tolerance index (DMSI) were calculated employing the formula used by Sen et al.^27^ and Raza et al.^28^,

$$\mathrm{PHSI}=\left(\frac{\mathrm{Height\, of\, stressed\, plants}}{\mathrm{Height\, of\, control\, plants}}\right)\times 100$$

$$\mathrm{RLSI}=\left(\frac{\mathrm{Root\, length\, of\, stressed\, plants}}{\mathrm{Root\, length\, of\, control\, plants}}\right)\times 100$$

$$\mathrm{SLSI}=\left(\frac{\mathrm{Shoot\, length\, of\, stressed\, plants}}{\mathrm{Shoot\, length\, of\, control\, plants}}\right)\times 100$$

$$\mathrm{DMSI}=\left(\frac{\mathrm{Dry\, matter\, of\, stressed\, plants}}{\mathrm{Dry\, matter\, of\, control\, plants}}\right)\times 100$$

### Assessment of biochemical content

After 25 days of the plantation, seedlings were uprooted, crushed, and used for estimation of biochemical contents.

#### Estimation of chlorophyll

The total chlorophyll content of the treated seedlings was assessed using the method of Arnon^29^.

#### Estimation of total carbohydrates (soluble sugars)

Wheat seedlings (0.1 g) were crushed in hot ethanol (80%) and a clear solution was obtained through filtration using Whatman No. 1 filter paper. Existing ethanol was evaporated by heating and aqueous reconstruction was made for the assay. Total soluble sugar content was determined by the Anthrone method^30^.

#### Estimation of total protein content

Total protein content was estimated using the methodology of Lowry et al.^31^. After mixing 5 mL alkaline copper solution and Folin-Ciocalteu reagent (FCR) with 1 mL seedling extract, the absorbance of the formed blue-colored complex was measured at 660 nm.

#### Estimation of total phenol content

With slight modification, the method prescribed by Kadam et al.^32^ was followed for total phenol content estimation. For that, distilled water (5 mL), 95% ethanol, 50% Folin-Ciocalteu reagent, and sodium carbonate (5%) was added to 1 mL sample and incubated for 1 h and the content was calculated using gallic acid standard.

## Quantitative estimation of stress-related parameters

### Estimation of free proline content

Free proline content was estimated following the method used by Bates et al.^33^. To obtain sample extract, leaves sample (0.5 g) was homogenized in 10 mL of 3% sulfosalicylic acid and centrifuged at 10,000 rpm for 8 min. Estimation was done by taking absorbance (520 nm) of the light pink colored toluene layer separated from the reaction mixture containing 2 mL acid ninhydrin, 2 mL glacial acetic acid, 1 mL leaf extract, and 4 mL of toluene.

### Determination of lipid peroxidation

The extent of lipid peroxidation was determined by measuring MDA contents following the protocol of Huang et al.^34^. Fresh leaf samples (fresh weight/FW-0.5 g) were crushed in 5 mL (Vt) 0.5% (w/v) TCA and centrifuged (cold centrifugation at 4 °C) for 10 min at 10,000 rpm. The reaction mixture containing an equal amount of sample (V1) and 0.6% (w/v) thiobarbituric acid (TBA) was heated for 30 min at 95 °C, followed by cold (ice water) treatment. The absorbance of the supernatant was measured at three different wavelengths viz*.* 450 nm (A_450_), 532 nm (A_532_) and 600 (A_600_) nm. The malondialdehyde (MDA) content was estimated using the following formula: MDA (μmol g^−1^ FW) = [6.45 × (A_532 _− A_600_) − 0.56 × A_450_] × Vt/(V1 × FW).

### Evaluation of plasma membrane integrity through electrolyte leakage estimation

After harvesting plant samples at 25th DAT, root samples (200 mg) of all the treatments were excised and placed in separate conical flasks containing 20 mL of deionized water. Subsequently, initial electrical conductivity (C_I_) was measured using a HANNA DiST3 HI98303 conductivity tester. Conical flasks were then placed in an orbital rotary shaker (Thermotech, L-7003) for 48 h, and after that electrical conductivity (C_N_) was measured again. After autoclaving the samples at 120 °C, final electrical conductivity (C_F_) was noted. Electrolyte leakage was estimated using the formula of Mosa et al.^35^.

$${\text{Electrolyte}}\;{\text{leakage}}\;\left( {{\text{E}}_{{\text{T}}} } \right) = \left( {\left( {{\text{C}}_{{\text{N}}} - {\text{C}}_{{\text{I}}} } \right)/{\text{C}}_{{\text{F}}} } \right) \times {\text{1}}00$$

### Histochemical detection of lipid peroxidation and plasma membrane integrity

The extent of lipid peroxidation was further detected histochemically using the methodology reported by Gupta and Mandal^36^. Roots were placed in Schiff’s reagent for the detection and incubated until the red colour was developed. Potassium sulphite solution (0.5% w/v, in 0.05 M HCl) was used to remove the extra stain, and images were taken by scanning the root samples in a digital scanner (Canon LiDE 110).

Histochemical detection of plasma membrane integrity was carried out according to the method prescribed by Yamamoto et al.^37^. Excisedroot was stained for 30 min using Evan’s blue solution (0.025% *w*/*v* dissolved in 100 mM CaCl_2_). The extra stain was removed by rinsing thrice with double distilled water and image acquisition was done by the same method as described above.

### Assessment of antioxidant enzymes

Leaf samples from each treatment (25th day after transplantation) were collected, cryo-crushed, and after cold-centrifugation at 10,000 rpm, the supernatant was used for further assay.

#### Estimation of catalase (CAT) activity

CAT (EC 1.11.1.5) activity was measured according to Hasanuzzaman et al.^38^. To 40 µL of enzyme, H_2_O_2_-potassium phosphate buffer (1:2) combination was mixed and decomposition of hydrogen peroxide was monitored at 240 nm. Enzyme activity was expressed as unit mg protein^-1^ (1 unit = mmole H_2_O_2_ reduced per minute) using an extinction coefficient of 39.4 M^−1^ cm^−1^.

#### Estimation of peroxidase (POD) activity

POD (EC 1.11.1.7) enzyme activity was determined using the method of Rani et al.^39^. To 3 mL of pyrogallol solution, 100 µL of the enzyme extract was added and the spectrophotometer was adjusted to read zero at 430 nm. H_2_O_2_ (0.5 mL) was directly added to the test cuvette and mixed well. The change in absorbance was recorded every 30 s up to 3 min. One unit of peroxidase is defined as the change in absorbance per minute at 430 nm and was expressed as U mL^−1^.

### Estimation of polyphenol oxidase (PPO) activity

PPO (EC 1.14.18.1) was estimated according to the method given by da Silva and Koblitz^40^ with some modifications. The reaction mixer contains 2.5 mL of potassium phosphate buffer (0.1 M, pH 6.5), 0.3 mL catechol solution (0.01 M), and 0.2 mL of enzyme extract. Absorbance was taken at 495 nm after 5 min of reaction. The activity was expressed as U mL^-1^, where one unit of PPO was defined as the amount of enzyme required for increasing unit absorption of the reaction mixture in each minute.

### Estimation of NADPH oxidase (NOX) activity

Determination of NOX (EC 1.6.3.1) was done by adding 0.4 mL of enzyme extract to the 2 mL of reaction mixture containing 50 mM Tris/HCl buffer (pH 7.5), 100 µM EDTA, 0.5 mM XTT, and 134 µM NADPH and absorbance was taken at 470 nm. An extinction coefficient of 21.6 mM^−1^ cm^−1^ was used to calculate enzyme activity^41^.

### Data analysis and program used

ImageJ (NIH Image) software was used to measure the grain size of the synthesized nanomaterials. Data of 30 replicates were taken into consideration for evaluation of morphological and biomass attributes and results were expressed as mean ± standard deviation (SD), however for biochemical, antioxidant enzymes, and stress-related parameters assessment, three replicates were taken. Tests of statistical differences were determined using Excel macros DSAASTAT, version 1.022 (DSAA, Italy) following Duncan’s multiple range test (DMRT) at *p* ≤ 0.05, where the applied treatments differ significantly were distinguished with different letters (a, b, c, etc.). Graphs were plotted using MS Excel and OriginPro 2021 (Origin Lab, USA) software. Heatmap was prepared using web-enabled Clustvis program^42^ based on the Pearson distance measurement method. Principal component analysis (PCA) was performed using OriginPro 2021 software and to make biplots PC1 and PC2 (first two components) were used.

## Results and discussion

### Characterization of the synthesized nanomaterials

**Figure 1:**
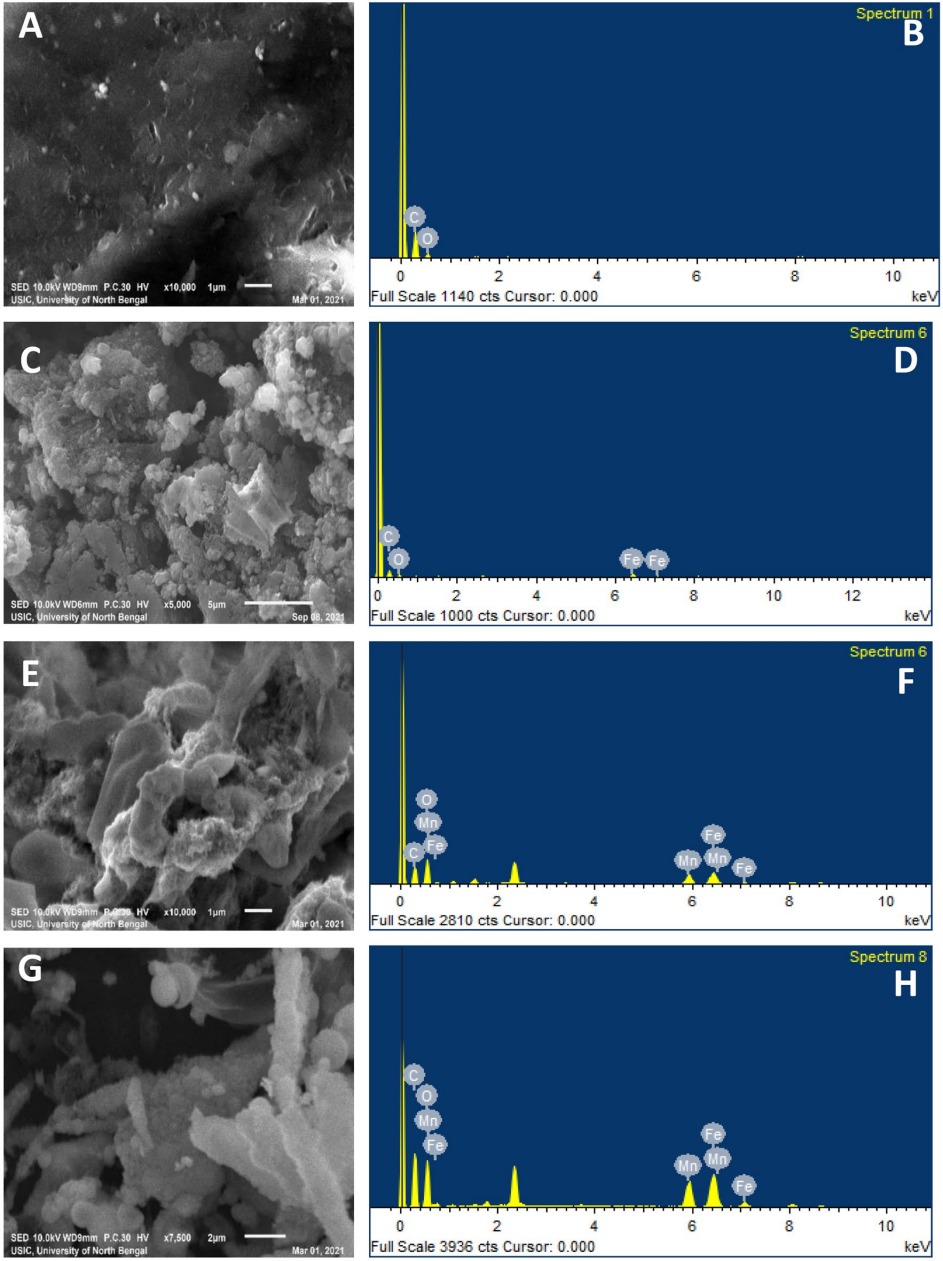
SEM micrograph (**A**,**C**,**E**,**G**) and EDS (**B**,**D**,**F**,**H**) elemental profile of GQD (**A**,**B**), iron nanoparticles (**C**,**D**), Fe–Mn nanocomposites (**E**,**F**) and Fe–Mn nanocomposites doped GQD (**G**,**H**).

**Figure 2:**
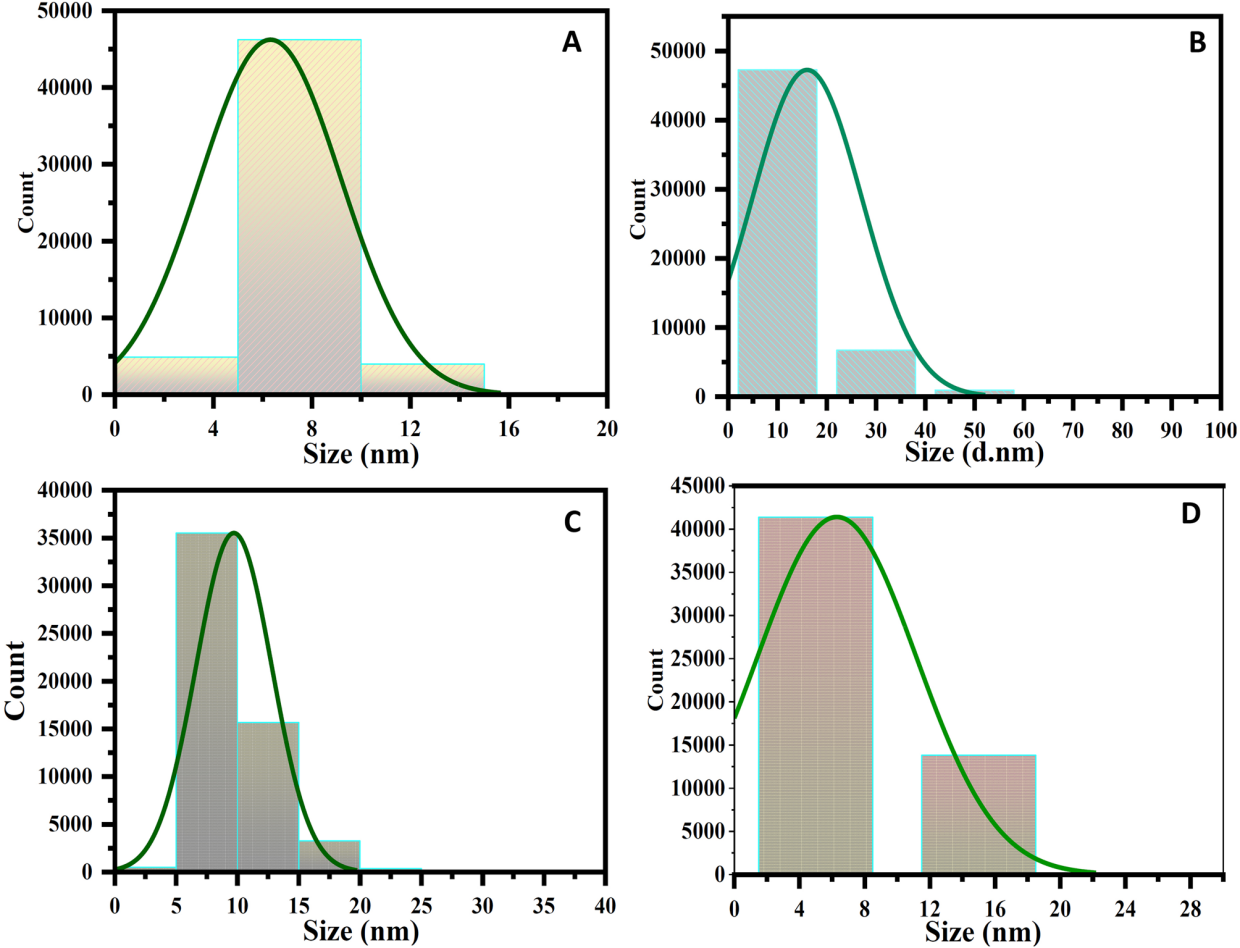
Particle size distribution of GQD (**A**), iron nanoparticles (**B**), Fe–Mn nanocomposites (**C**) and Fe–Mn nanocomposites doped GQD (**D**).

Figure 1A,C,E,G showed the surface morphology of GQD, FeNP, Fe–Mn nanocomposites, and Fe–Mn nanocomposites doped GQD respectively as observed through SEM. There is a significant difference in morphology among the synthesized samples, which may be attributed to the good incorporation of the metal oxides on the GQD sheets. The particle size of this nanomaterial was analyzed using ImageJ software and the size distribution was presented in Fig. 2A–D. As displayed in Fig. 1A the surface of GQD is looking like a highly broken sheet structure with average size of 5.09 nm. Apart from GQD the surface morphology of Fe–Mn nanocomposites was found to be broken brick-like with an average size of 10.13 nm shown in Fig. 2C. The surface morphology of Fe–MN nanocomposites doped GQD was totally different which may be attributed to the high incorporation of metal oxide into the GQD nano-sheet. The average size of the synthesized nanocomposites was found to be 5.26 nm with spherical particles deposited on the GQD. The dominant count rate of the particle size is in the range of 5–15 nm, confirming the formation of nanocomposites.

The elemental composition of the prepared nanomaterials was determined by EDS elemental profiling. Figure 1B,D,F,H displayed the EDAX spectrum GQD, FeNP, Fe–Mn nanocomposites, and Fe–Mn nanocomposites doped GQD respectively. The entire spectrum shows that the prepared composites only contain C, Fe, Mn, and O which are expressed with atomic and weight percentages and presented in Table 2.

**Table 2:** Tabular format of elemental composition of synthesized nanomaterials as observed EDS.

Nanomaterials	Element	Series	App conc	Intensity corn	Weight%	Weight% sigma	Atomic%
GQD	C	K	11.30	1.2962	67.90	4.45	73.80
	O	K	1.16	0.2819	32.10	4.45	26.20
F_NP	C	K	0.75	0.8836	51.06	5.07	67.71
	O	K	0.24	0.5726	25.80	4.90	25.69
	Fe	K	0.31	0.8216	23.14	3.24	6.60
Fe–Mn nano-composites	C	K	7.61	0.7803	5.72	0.56	9.76
	O	K	12.55	0.5071	16.35	0.54	6.64
	Mn	K	13.33	0.8381	40.55	1.30	41.56
	Fe	K	15.50	0.8514	37.39	1.36	42.04
Fe–Mn nano-composite doped GQD	C	K	21.61	0.7803	38.72	1.56	49.76
	O	K	17.55	0.5071	48.35	1.54	46.64
	Mn	K	3.33	0.8381	5.55	0.30	1.56
	Fe	K	4.50	0.8514	7.39	0.36	2.04

*GQD* graphene quantum, *F_NP* iron nanoparticles.

**Figure 3:**
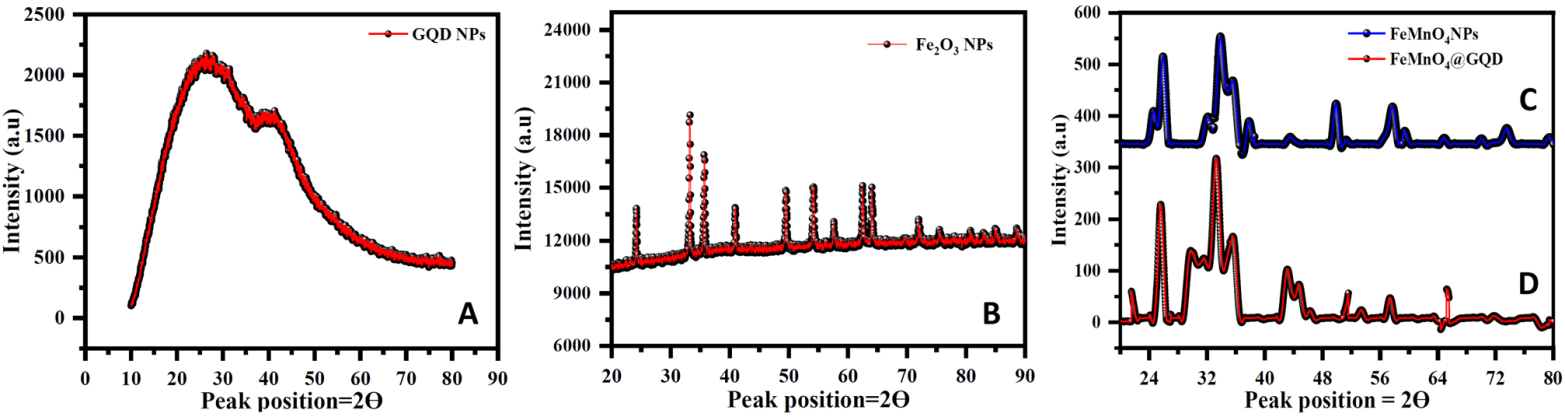
XRD spectra of GQD (**A**), iron nanoparticles (**B**), Fe–Mn nanocomposites (**C**) and Fe–Mn nanocomposites doped GQD (**D**).

The crystallinity and structure of the synthesized nanomaterials were investigated using an X-ray diffraction study (XRD) and the obtained spectra were presented in Fig. 3A–D. The XRD spectra of GQD (Fig. 3A) showed a broad peak at 2θ = 23.68° with an interplanar spacing of 0.34 nm, which corresponds to (002) planes and a peak centered at 40° for corresponding to 101 planes. All peak profiles of GQD XRD patterns matched with JCPDS card No.-75-0444. The broad peak observed at 2θ = 24° might be due to the small size effect of the GQDs, and the larger interlayer distance of the GQDs may be ascribed to the presence of oxygen functional groups^43^. Figure 3B depicted the X-ray diffraction (XRD) pattern of iron oxide nanoparticles. The most intense peak of the XRD pattern of prepared material was at 32.95° corresponding to (104) plane, confirming the formation of the α-Fe_2_O_3_ phase. Each peak observed in the XRD pattern (can be indexed to 2θ = 23.34, 32.95, 35.14, 40.73, 49.54, 54.23, 63.12, 65.45, 71.23 etc. corresponds to 012, 104, 110, 113, 024, 116, 018, 214, and 300 miller indexes) represents the α-Fe_2_O_3_ structure having hexagonal configuration (JCPDS Card No. 86–0550). Figure 3C displayed the XRD peak profile of Fe–Mn nanocomposites, the peak appeared at 2θ = 24, 33, 36, 41, and 48 are directly related to iron oxide with miller indexes (012) (104) (110) and (024), the peak profile matches with JCPDS card no.-33-0664. The peak appeared at 2θ = 71, 61, 50, 42, 38, 29, and 18 are directly related to the α-MnO_2_ structure with miller indexes (200), (541), (521), (411), (301), (211) and (310) which were in agreement with the JCPDS card no.-044-0141. The diffraction angles for Fe–Mn nanocomposites doped GQD shown in Fig. 3D, centered at 18.16, 20.287, 21.044, 24.153, 25.605, 28.468, 33.52, 35.136, 44.789, and 49.329 corresponds to the miller index to crystal planes (100), (101), (102), (012), (103), (200), (104), (110), (202) and (024), respectively. The obtained XRD patterns matched with file no. 00-024-0072 and appeared to be hexagonal hematite structure. It was noted that a small diffraction peak was observed at around 26.38. It is believed that the peak shifting could be attributed to disordered structures on the edge of GQDs^44^. Moreover, the peak was not observed in the non-GQDs sample i.e. Fe–Mn nanocomposites. Therefore, it was believed that the existence of GQDs related peaks indicated the formation of Fe–Mn nanocomposites doped GQD.

**Figure 4:**
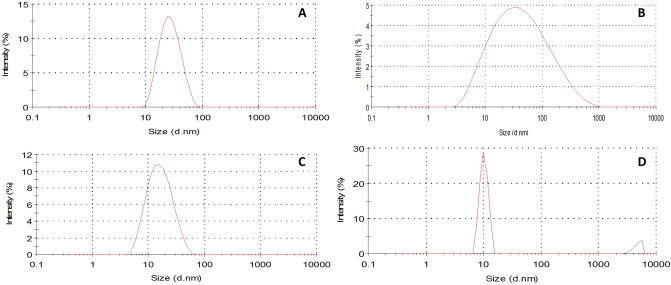
DLS size distribution pattern of GQD (**A**), iron nanoparticles (**B**), Fe–Mn nanocomposites (**C**), and Fe–Mn nanocomposites doped GQD (**D**).

Figure 4A–D represented the DLS spectra of GQD, FeNP, Fe–Mn nanocomposites and Fe–Mn nanocomposites doped GQD, and the average hydrodynamic size of the corresponding nanomaterials is found to be 28, 65, 10, and 17 nm respectively. The average hydrodynamic size of the composite materials was somewhat higher than the size measured from SEM, this might be due to some extent of hydration of the surface of Fe-MnO_4_ nanocomposites doped GQD in an aqueous solution.

### Effects of nanocomposites on wheat seed germination

The process of germination is conceptually simple but is of crucial process of prime significance that eventually influences crop yield and quality. The present study reveals that almost all the germination-related parameters such as germination percentage, germination index (GI), mean germination time (MGT), coefficient of velocity of germination (CVG) etc. were found to be improved over control on application of nanocomposites as treatment solution during solid matrix priming (Table 3). Control unprimed (C_UP) seeds required significantly more days for germination than the simple hydroprimed seeds (C_HP), indicating the ameliorative role of solid matrix priming in the speed of germination^45^. MGT which measures the rate and time-spread of germination indicated that FM_GQD_5 and FM_NC_2 took only 33.5 h and 34.2 h (average) respectively to germinate all of the viable seeds which were 16.91 and 15.17 percent less than the control unprimed seeds (C_UP need 40.32 h and C_HP need 36.79 h). However, no statistical difference (*p* ≤ 0.05) was observed among hydroprimed and nanoprimed seeds in MGT attribute. As compared to C_UP (control unprimed) seeds, the germination percentage was significantly higher in nano-primed seeds, especially in the case of iron-manganese doped graphene quantum dots at 500 µg/mL which showed 135% improvement over control. Higher dosage of iron nanoparticles (F_NP_5) and low dose of iron-manganese nanocomposites (FM_NC_2) showed the same kind of results in terms of germination percentage. Also, among control unprimed and hydroprimed seeds, no statistical difference was observed. Germination index (GI), which is the evaluation of the percentage and speed of germination, indicates a marked difference between various treatments and control (i.e. C_HP and C_UP)^46^. Highest germination index was observed at FM_GQD_5 treatment, followed by FM_NC_2 and F_NP_5. As higher values of GI indicates a greater rate of germination, the effects of applied nano-materials on the germination of wheat seeds were highly pronounced from the observed results^47^. In the contrary, promptness index (PI) showed no statistical difference among control unprimed, hydroprimed, and higher dosage of normal iron nanoparticles and graphene quantum dots (i.e. F_NP_5 and GQD_5) primed seeds. However, our nano-molecule of interest i.e. iron-manganese nanocomposites doped graphene quantum recorded the highest for PI, which exhibited 84% improvement over control. The coefficient of velocity of germination (CVG) depicted the rapidity of germination^48^. Value of CVG depends on the number of seeds germinated and the time taken for germination and its value increases when more seeds germinated in less time. From the experimental results, it was observed that FM_GQD_5 showed the highest CVG value, and increased by 21.32 and 19.20% over control-unprimed and hydroprimed treatments respectively. On summarizing the results of different germination-related parameters, it can be said that seed priming has a primary role in germination improvement as control hydroprimed showed better performance than the unprimed seeds. On the other side priming effect with nano solution (nanopriming) exhibited comparatively higher germination capability than the normal hydroprimed seeds and among nanoprimed, seeds primed with iron-manganese nanocomposites doped GQD marked highest for the tested germination-related parameters and indices.

**Table 3:** Various types of germination-related parameters of the wheat seeds primed with different treatment solutions. Results are expressed as mean ± SD. Values with different letters (a, b, c, etc.) differ significantly at *p* ≤ 0.05 by Duncan’s multiple range test (DMRT) test.

Treatments	Attributes				
	Germination percentage (GP)	Germination index (GI)	Promptness index (PI)	Mean germination time (MGT)	Coefficient of velocity of germination (CVG)
GQD_2	65.484 ± 4.623^b^	74.500 ± 1.887^c^	90.363 ± 3.562^b^	1.443 ± 0.057^a^	2.863 ± 0.071^ab^
GQD_5	40.390 ± 3.975^d^	47.833 ± 2.566^e^	68.677 ± 4.904^d^	1.513 ± 0.050^a^	2.740 ± 0.056^b^
FM_GQD_2	38.789 ± 4.466^d^	46.750 ± 2.500^ef^	72.347 ± 5.956^cd^	1.533 ± 0.050^a^	2.733 ± 0.068^b^
FM_GQD_5	78.687 ± 3.501^a^	93.000 ± 1.887^a^	119.873 ± 4.212^a^	1.397 ± 0.050^a^	2.997 ± 0.120^a^
FM_NC_2	72.944 ± 3.308^ab^	83.500 ± 2.000^b^	95.713 ± 4.368^b^	1.427 ± 0.065^a^	2.947 ± 0.162^ab^
FM_NC_5	54.936 ± 4.886^c^	63.500 ± 3.132^d^	79.313 ± 4.102^c^	1.440 ± 0.060^a^	2.967 ± 0.115^ab^
F_NP_2	50.639 ± 5.096^c^	58.667 ± 2.765^d^	66.030 ± 4.895^d^	1.503 ± 0.065^a^	2.767 ± 0.091^ab^
F_NP_5	70.158 ± 4.385^ab^	83.667 ± 2.268^b^	115.420 ± 4.53^a^	1.497 ± 0.080^a^	2.820 ± 0.115^ab^
C_HP	40.300 ± 3.368^d^	46.917 ± 2.373^e^	73.273 ± 3.781^cd^	1.533 ± 0.065^a^	2.743 ± 0.087^b^
C_UP	33.441 ± 4.118^d^	41.000 ± 2.250^f^	64.900 ± 3.831^d^	1.683 ± 0.065^b^	2.470 ± 0.095^c^

*GQD_2* graphene quantum dots 200 µg/mL, *GQD_5* graphene quantum dots 500 µg/mL, *FM_GQD_2* Fe–Mn nanocomposites doped graphene quantum dots 200 µg/mL, *FM_GQD_5* Fe–Mn nanocomposites doped graphene quantum dots 500 µg/mL, *FM_NC_2* Fe–Mn nano-composites 200 µg/mL, *FM_NC_5* Fe–Mn nano-composites 500 µg/mL, *F_NP_2* iron nanoparticles 200 µg/mL, *F_NP_5* iron nanoparticles 500 µg/mL, *C_HP* control hydroprimed; *C_UP* control unprimed non-saline.

Previously, some carbon-based nanomaterials such as carbon nanotubes, and carbon nanoparticles have been reported for their role in seed germination improvement of various vegetables and crops even under salinity^49,50^. Indeed, seed priming is one of the most promising techniques used for stress tolerance induction and enhancement of crop yield in plants^51^. In the last few years, the nanopriming method using synthetic nanoparticles gained significant importance in crop advancement owing to their distinctive physicochemical properties and small size^52^. Besides improving plant growth, nanoparticles also safeguard plants from various kinds of stresses. According to the publised report, the accessibility of micronutrients like iron and manganese in the seeds is vital for the synthesis of protein and enzymes responsible for seedlings to efficiently utilize the other nutrients in the soil^53^. Some other reports suggested that there was a strong influence of external carbon/nitrogen ratio in the regulation of seed dormancy, germination of seeds, and seedling development^54^. In the present study improvement of germination parameters observed by the application of graphene quantum dots and iron-manganese nanocomposites through solid matrix priming could be due to the role of carbon and other micronutrients (Fe, Mn) in the germination process. Besides that, seed pre-treatments with nanomaterials facilitated the germination process by availing earlier uptake of treatment solutions^53^.

### Effects of nanocomposites on wheat seedling growth under salinity

**Figure 5:**
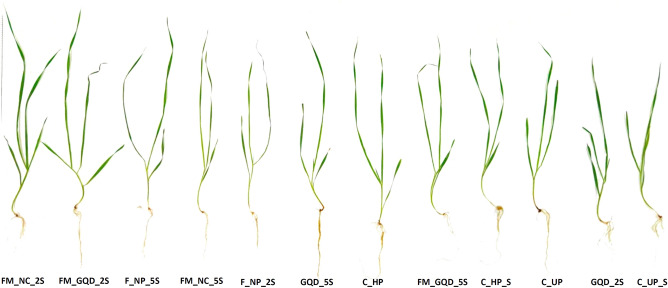
Phenotypic appearance of the treated wheat seedling. *GQD_2* graphene quantum dots 200 µg/mL, *GQD_5* graphene quantum dots 500 µg/mL, *FM_GQD_2* Fe–Mn nanocomposites doped graphene quantum dots 200 µg/mL, *FM_GQD_5* Fe–Mn nanocomposites doped graphene quantum dots 500 µg/mL, *FM_NC_2* Fe–Mn nano-composites 200 µg/mL, *FM_NC_5* Fe–Mn nano-composites 500 µg/mL, *F_NP_2* iron nanoparticles 200 µg/mL, *F_NP_5* iron nanoparticles 500 µg/mL, *C_HP* control hydroprimed, *C_UP* control unprimed non-saline.

**Figure 6:**
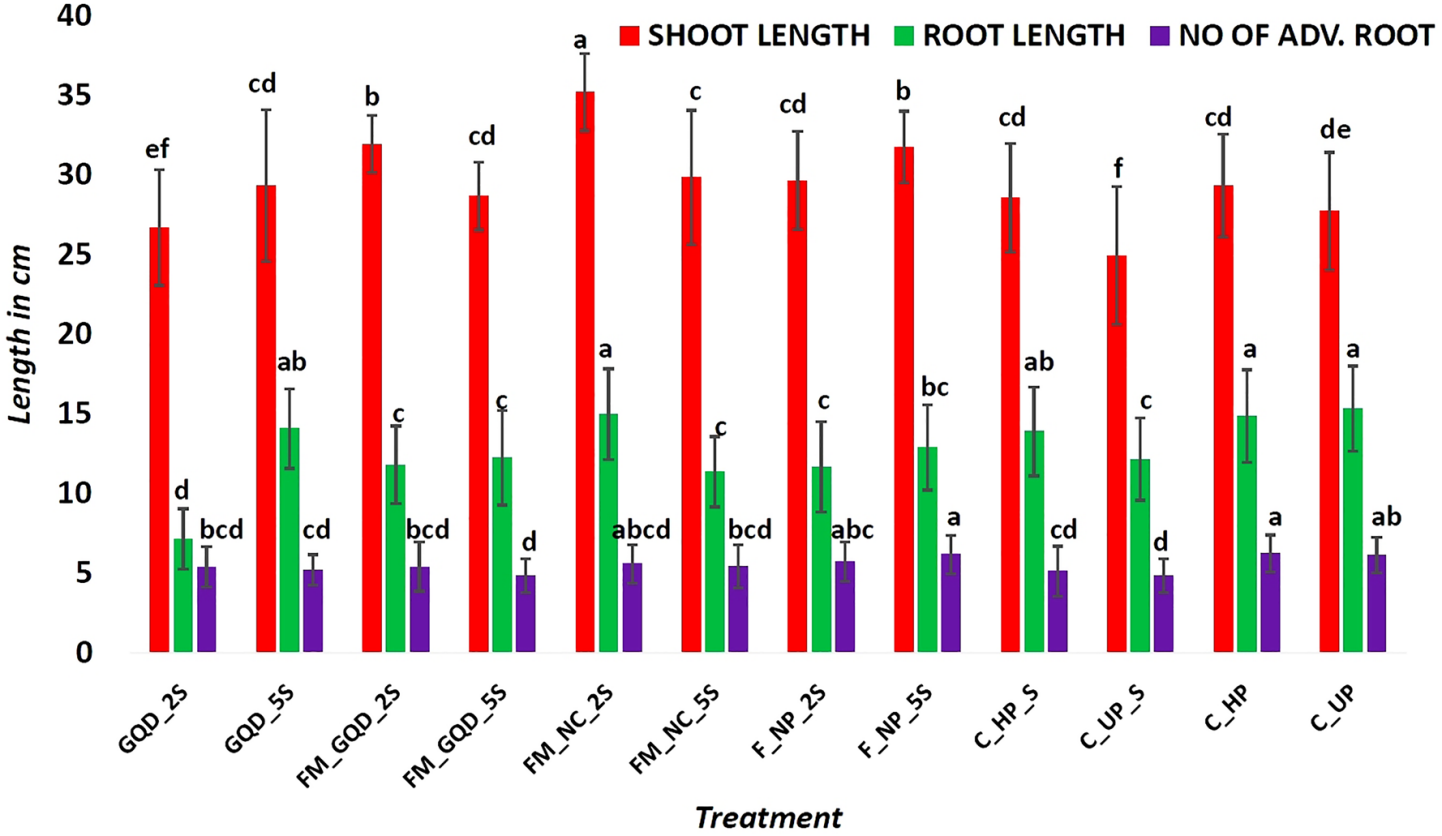
Shoot length, root length, and the number of adventitious roots of the treated wheat seedling. Each vertical bar represents the standard deviation. Whereas, treatments with different letters (a, b, c, etc.) differ significantly at *p* ≤ 0.05 by Duncan’s multiple range test (DMRT) test.

Soil salinity has detrimental effects on various morphological attributes of wheat plants including seedling growth, root length, root numbers, root/shoot ratio, leaf area, biomass, and chlorophyll content. Uniformly germinated seedlings with approximately 2 cm coleoptile were transferred onto a sand bed under NaCl-mediated salinity to examine the effects of applied nano-material on post-germination seedling growth. The morphological appearance of the treated wheat seedlings was presented in Fig. 5. Low concentration of Fe–Mn nanocomposites (FM_NC_2S) was the most effective treatment for root length (14.98 cm) which was almost similar to that of control unprimed and hydroprimed non-saline treatments while shortest roots were formed in the GQD_2S (7.15 cm). Shoot length was enhanced by 41.13, 28.06, and 27.26 percent in FM_NC_2S, FM_GQD_2S, and F_NP_5S treatments respectively as compared to salinity imposed control unprimed seeds (C_UP_S) (Fig. 6). On evaluating a number of the adventitious root, it was found that except for control hydroprimed non-saline (C_HP) and F_NP_5S treatments, there was no significant distinction among different treatment solutions and control groups. Improvement of root number as up to control hydroprimed (non-saline) by application of iron nanoparticles may be due to the formation of adventitious roots is controlled by various morpho-physiological processes and generally facilitated by several external factors including mineral nutrition. Past reports indicated the accumulation of several nutrients including iron, copper, and manganese at the stem base^55,56^.

**Figure 7:**
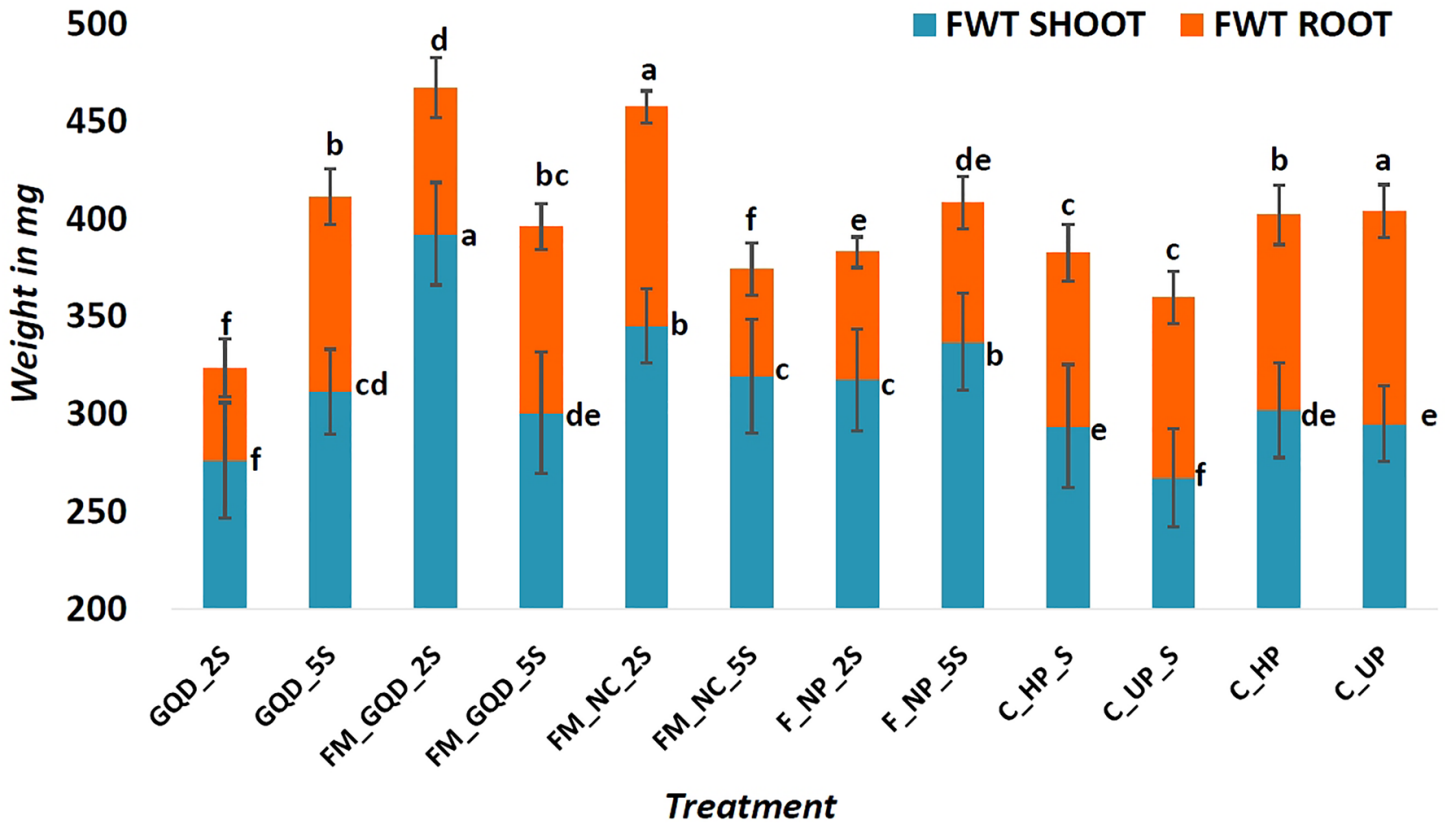
Fresh weight (FWT) of shoot and root of the treated wheat seedling. Each vertical bar represents the standard deviation. Whereas, treatments with different letters (a, b, c, etc.) differ significantly at *p* ≤ 0.05 by Duncan’s multiple range test (DMRT) test.

**Figure 8:**
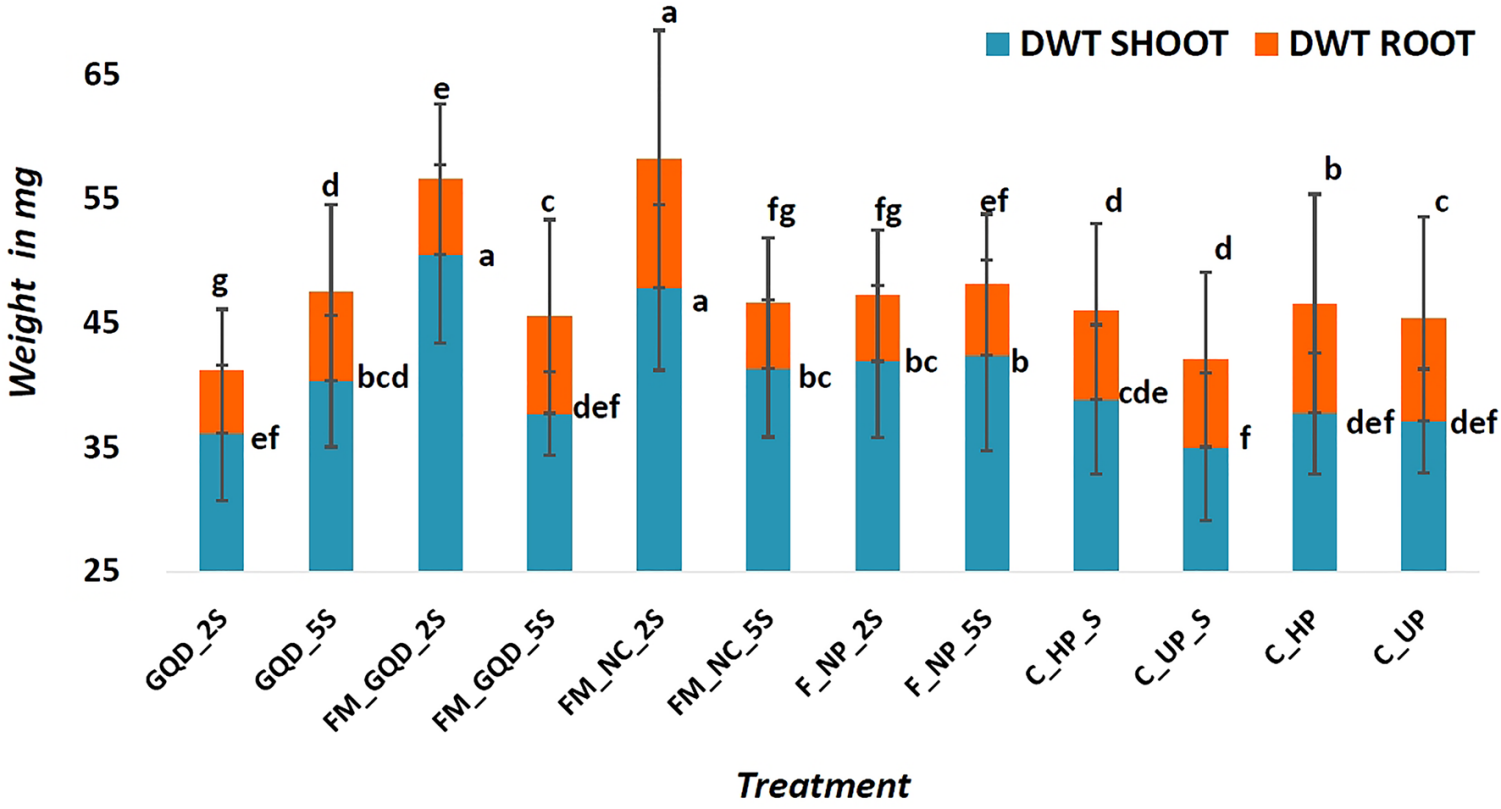
Dry weight (DWT) of shoot and root of the treated wheat seedling. Each vertical bar represents the standard deviation. Whereas, treatments with different letters (a, b, c, etc.) differ significantly at *p* ≤ 0.05 by Duncan’s multiple range test (DMRT) test.

An increase in fresh as well as dry weight of both shoot and root was also observed in nanoprimed especially iron-manganese doped graphene quantum dots and iron-manganese nanocomposites primed seedlings compared to unprimed seedlings (Figs. 7 and 8). FM_GQD_2S showed improvement in both fresh and dry weight over the total control group (i.e. saline as well as non-saline). These results indicated that the incorporation of iron-manganese nanocomposites inside graphene quantum dots remarkably improved seedling's vigour as compared to unprimed seeds. Whereas FM_NC_2S recorded the highest for fresh and dry weight of root which correlated with root length data and in comparison to unprimed seed, this particular treatment showed an improvement of 21.77% and 47.57% for fresh and dry weight respectively.

In the present study, seeds primed with nanocomposites emerged earlier in comparison to the control, which might have increased their establishment resulting in superior use of applied nutrient solutions as evidenced by better shoot length, root length, and seedling biomass. In addition to that, an increase in the root length of seeds primed with iron nanoparticles (F_NP_S) could be due to the activation of cell cycling and respiration during priming. Activation of cell cycling and respiration, translocation of assimilated materials, and weakening of seed coat structure leads to faster root emergence^57,58^.

### Assessment of stress tolerance index

**Figure 9:**
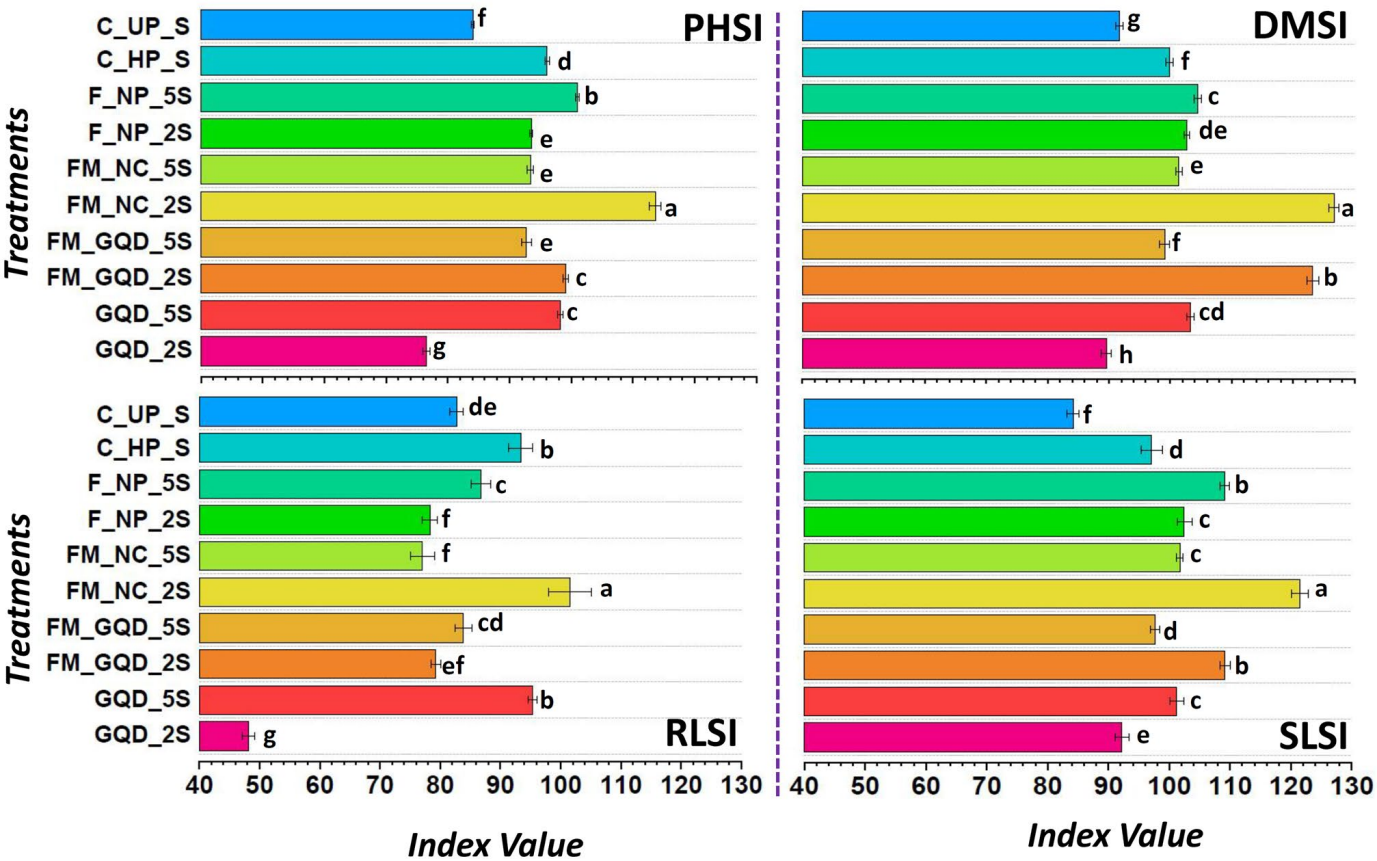
Graphical expression of different types of stress tolerance index (*PHSI* plant height stress tolerance index, *DMSI* dry matter stress tolerance index, *RLSI* root length stress tolerance index, *SLSI* shoot length stress tolerance index). Each horizontal bar represents the standard deviation. Whereas, treatments with different letters (a, b, c, etc.) differ significantly at *p* ≤ 0.05 by Duncan’s multiple range test (DMRT) test.

Several stress tolerance indices are taken into consideration, aiming to assist identification and selection of stable treatments that have enough capability to fight with the imposed stress (salinity). Among different treatments solution, seedlings treated with FM_NC_2S (iron-manganese nanocomposites at 200 µg/mL) showed maximum value for all the studied stress tolerance indexes. In comparison to the control, this particular treatment has enhanced plant height stress tolerance index (PHSI), dry matter stress tolerance index (DMSI), root length stress tolerance index (RLSI), and shoot length stress tolerance index (SLSI) by 35, 38.14, 22.73, and 44.18% respectively (Fig. 9). Whereas iron-manganese doped graphene quantum dots at the same dose (FM_GQD_2S) stood in second position and showed 34.38 and 29.69% improvement over control for DMSI and SLSI respectively.

### Assessment of biochemical content

**Figure 10:**
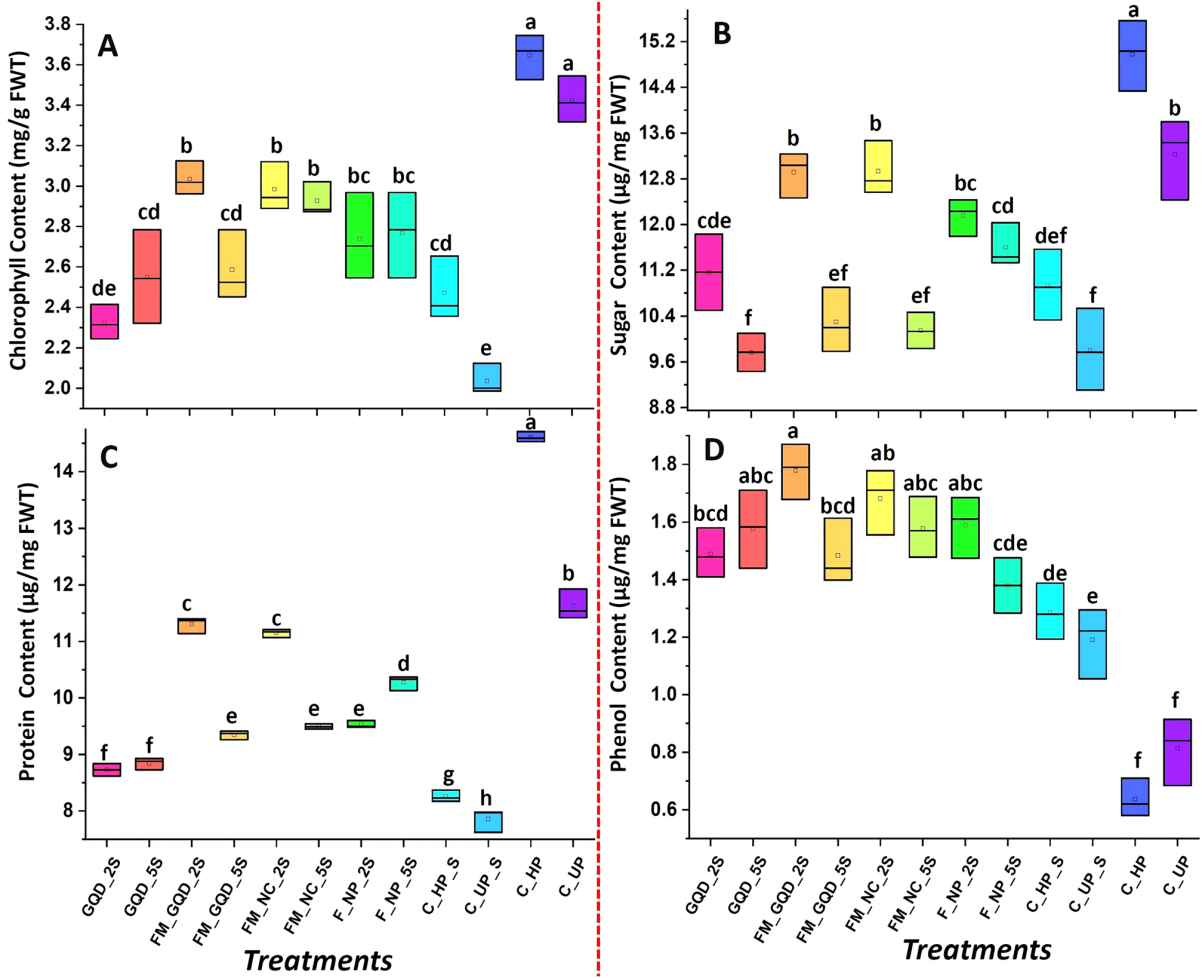
Box plot represent the effect of applied nanomaterials on different biochemical parameters (**A** chlorophyll content, **B** total soluble sugar content, **C** protein content, **D** total phenol content). Treatments with different letters (a, b, c, etc.) differ significantly at *p* ≤ 0.05 by Duncan’s multiple range test (DMRT) test).

A plant needs optimum photosynthesis for its normal growth and survival, which is greatly influenced by its surroundings environment^59^. Salinity stress imposed an adverse effect on photosynthetic activity by destroying chlorophyll and inhibiting the PSII (photosystem II) activity. High salt stress inhibits photosynthesis by the accumulation of Na^+^ and Cl^−^ ions in the chloroplast and by the reduction in the water potential of plant^60^. Guo et al.^61^ observed that in the wheat plant, salinity stress reduced CO_2_ absorption and transpiration rate and induced stomatal closure, which eventually caused a serious reduction in the final productivity. In this study, chlorophyll content declined in both control and treated wheat seedling after salt stress application in respect to the control non-saline group. But in terms of chlorophyll, total soluble sugar, and protein content, the control saline group is more affected than the salinity-imposed nano-treated seedling (Fig. 10).

From the experimental result, it was observed that being highest among the salinity imposed group there was no variation among the FM_GQD_2S and both the dosage of iron-manganese nanocomposites (FM_NC) in terms of chlorophyll content. A wide number of research works reported that the foliar application of nanoparticles dramatically improved the chlorophyll contents of plants, by synthesizing more light-harvesting complexes to absorb maximum light energy for improved photosynthesis^62,63^. According to previous studies that have examined the effect of nanoparticles on salt-stressed plants, most of the available nanoparticles were able to amplify photosynthesis by increasing the photosynthetic pigments content^62,64–67^. Improvement of chlorophyll content in Fe–Mn nanocomposites and composite doped graphene quantum may be due to iron and manganese are two vital components in photosynthetic cells. Iron has an immense role in the synthesis of cytochrome and several other heme-containing molecules as well as chlorophyll. Iron also influences the construction of Fe–S clusters which in turn transfer electrons in the photosystem through Fe^+2^/Fe^+3^ oxidation states^68^. In addition to that, manganese is found to be involved in different parts of the cell (in chloroplasts mitochondria, the structure of some enzymes etc.), maintain optimal photosynthetic rate even under stress condition, enhance the rate of electron transport and oxygen evolution during photosynthesis^69,70^. Shahi and Srivastava^71^ reported that, under salt stress, manganese supplementation to *Vigna radiata* caused an improvement in chlorophyll content, membrane stability index, and nitrate reductase activity. FM_GQD_2S and FM_NC_2S showed almost the same amount of total soluble sugar content as control unprimed non-saline seedlings, indicating a strong effect of iron-manganese nanocomposites and nanocomposites doped graphene quantum dots in the maintenance of sugar content even in saline condition. After salinity treatment protein content was decreased by 32.33% in control unprimed seedlings whereas nanocomposites doped GQD (FM_GQD_2S) showed only a 2.83% reduction from control unprimed non-saline one. The protein content of leaves is vastly critical and plays a vital role in the reproduction, growth, and eventual grain yield of plants^72^. Some reports suggested that under saline conditions protein quantity could be increased, but in the case of wheat and triticale it was decreased^2^. Decrease in protein content in leaves in response to salinity is not surprising as it is well established that ROS initially targets proteins in the biological system. On reaching chloroplast, ROS creates alteration in different proteins such as stromal and thylakoid, including inactivation and degradation of Rubisco^73,74^. Therefore, reduction in the protein content of the leaf is one of the vital indicators which is affected by salinity stress^75^. According to the report of Wan et al.^76^, the application of carbon nano-horns on *Sophora alopecuroides* seedlings through foliar spray under salt stress promoted fresh root biomass, leaf soluble sugar, and leaf and root total protein contents of the plants. Shafiq et al.^77^ experienced that wheat seeds pre-soaking with polyhydroxy fullerene nanoparticles remediated salinity stress in wheat seedlings through an increase in the production of amino acids, sugars, and K^+^ and P contents.

Phenolics are the most widely distributed secondary metabolites in the plant kingdom and play numerous roles in signaling, auxin transport, plant defense, and free radical scavenging^78^. Among various non-enzymatic antioxidants, phenols, and flavonoids by accumulating in various tissues of plants significantly act as free radicals scavengers for tolerating salt stress^79^. Salt stress caused an increase in total phenol content in the leaves, while maximum content was observed in the FM_GQD_2S treatment. On the other hand, there was no significant difference in total phenol content was observed among the remaining treatments. An increase in total phenol content of wheat seedlings by salt stress in comparison to control was previously reported by Kiani et al.^72^. Over the past decades, various researchers consistently reported the strong association between abiotic stress tolerance and polyphenols through maintenance of proper redox state in cells^80^.

### Quantitative estimation of stress-related parameters

Proline, the major stress indicator is an amino acid and compatible solute that generally accumulates in plants in response to various stress conditions including salinity^81^. Under various environmental stresses, proline is produced from glutamate due to the inefficiency of feedback regulation of the proline biosynthetic pathway^82^. Proline not only serves as an osmotolerant but also acts as a substrate for respiration and as a source of nutrients including nitrogen and other metabolites^83,84^. The overall results of the current study indicate that the entire seedling grown under saline condition maintained a higher proline level than those grown under normal (non-saline) condition (Table 4). However, a significantly higher quantity of proline accumulation was noticed in nano-treated seedlings when compared with the controlled saline group. FM_GQD_2S treated seedlings showed the highest proline accumulation which was 97.26 and 57.26% more than the control unprimed seedlings under non-saline (C_UP) and saline condition (C_UP_S) respectively. Increased accumulation of this kind of osmotolerant like proline has been reported to enhance salt tolerance^72^. Like other osmolytes, proline modulates redox potential by osmotic adjustment thereby stabilizing various enzymes and protecting cellular components from abiotic stresses^85^. Cell membrane is one of the primary targets of various environmental stresses. Maintenance of cell membrane stability and integrity is a sign of stress tolerance^86^. Accumulation of ROS due to stress caused oxidation of unsaturated fatty acid (the major membrane lipids) which induces lipid peroxidation and membrane degradation^87^.

**Table 4:** Different types of stress-related parameters of the treated wheat seedling. Results are expressed as mean ± SD. Values with different letters (a, b, c, etc.) differ significantly at *p* ≤ 0.05 by Duncan’s multiple range test (DMRT) test.

Treatments	Attributes		
	Proline content (µmol/g FWT)	MDA content (µmol/g FWT)	Electrolyte leakage (EL)
GQD_2S	3.736 ± 0.115^bc^	13.400 ± 0.721^bc^	32.545 ± 0.696^d^
GQD_5S	3.264 ± 0.199^de^	14.342 ± 0.684^bc^	35.533 ± 1.386^c^
FM_GQD_2S	4.327 ± 0.198^a^	9.306 ± 0.867^f^	25.659 ± 1.305^e^
FM_GQD_5S	4.020 ± 0.157^ab^	11.263 ± 0.916^de^	27.241 ± 0.900^e^
FM_NC_2S	4.240 ± 0.131^a^	10.336 ± 0.688^ef^	30.555 ± 0.962^d^
FM_NC_5S	3.665 ± 0.195^bcd^	12.639 ± 0.880^ cd^	32.441 ± 1.051^d^
F_NP_2S	3.624 ± 0.220^bcd^	12.987 ± 1.001^bcd^	37.159 ± 0.920^bc^
F_NP_5S	3.346 ± 0.226^cde^	13.628 ± 0.712^bc^	37.581 ± 1.581^bc^
C_HP_S	3.083 ± 0.208^ef^	14.650 ± 0.862^b^	39.224 ± 1.084^ab^
C_UP-S	2.747 ± 0.203^f^	16.466 ± 0.713^a^	40.372 ± 1.080^a^
C_HP	1.907 ± 0.194^g^	6.570 ± 0.901^g^	9.454 ± 0.944^f^
C_UP	2.193 ± 0.177^g^	7.468 ± 0.793^g^	10.220 ± 1.050^f^

*GQD_2* graphene quantum dots 200 µg/mL, *GQD_5* graphene quantum dots 500 µg/mL, *FM_GQD_2* Fe–Mn nanocomposites doped graphene quantum dots 200 µg/mL, *FM_GQD_5* Fe–Mn nanocomposites doped graphene quantum dots 500 µg/mL, *FM_NC_2* Fe–Mn nano-composites 200 µg/mL, *FM_NC_5* Fe–Mn nano-composites 500 µg/mL, *F_NP_2* iron nanoparticles 200 µg/mL, *F_NP_5* iron nanoparticles 500 µg/mL, *C_HP* control hydroprimed; *C_UP* control unprimed non-saline.

Malondialdehyde (MDA) content of seedlings acts as an indicator of the extent of lipid peroxidation in studies related to redox signaling and oxidative stresses, especially in experiments related to plant responses to abiotic and biotic stresses^88^. Present study reflects that salt stress caused an increase in MDA production in both control and treated wheat seedlings. However, under saline condition, the extent of lipid peroxidation is least in FM_GQD_2S (MDA content-9.306 ± 0.867µmolg^−1^ FWT) which is 43.48% less than the control. Indeed, the accumulation of lower MDA content signifies the higher antioxidative ability of a treatment solution and reflects higher ameliorative effects against salinity (Table 4). Various metal and metalloid-based nanoparticles are reported to increase salt tolerance by lowering the MDA level and through proper osmoregulation^89^. Liu et al.^90^ observed 44% less MDA accumulation by application of poly acrylic acid coated nanoceria with an oxidase-like activity, which plays an ameliorating role in cotton plants under saline condition.

As expected, the results of the present study showed a significant increase in electrolyte leakage (EL) percentage in response to salt stress (Table 4). However, a significant distinction was observed for the EL% response among different treatments under salt stress; with the highest values noted in control unprimed (C_UP_S) wheat seedlings (40.37%) and FM_GQD_2S showed the lowest (25.65%). Maintenance of plasma membrane integrity in a proper way is an important adaptive strategy of plants against free radicals^75,91^. In the present study, a higher electrolyte leakage percentage was found in control seedlings than in the treated under saline condition. These findings indicated that the plasma membrane may represent a promising strategy in the regulation of transmembrane ion and metabolite fluxes during stress conditions. The result of the current study is in good agreement with those reported by Radi et al.^92^, who observed an increase in electrolyte leakage percentage in wheat due to salinity stress and also experienced that the salt-sensitive cultivar had higher EL% than the salt-tolerant one. Lower electrolyte leakage in iron-manganese doped graphene quantum dots can be correlated with a high amount of total phenol content and less lipid peroxidation as all of these phenomena are interactive. According to previous reports, phenolic compounds play a vital role in quenching singlet oxygen, H_2_O_2_-scavenging, and reducing or inhibiting lipid oxidation^93,94^.

### Histochemical detection of lipid peroxidation and plasma membrane integrity

**Figure 11:**
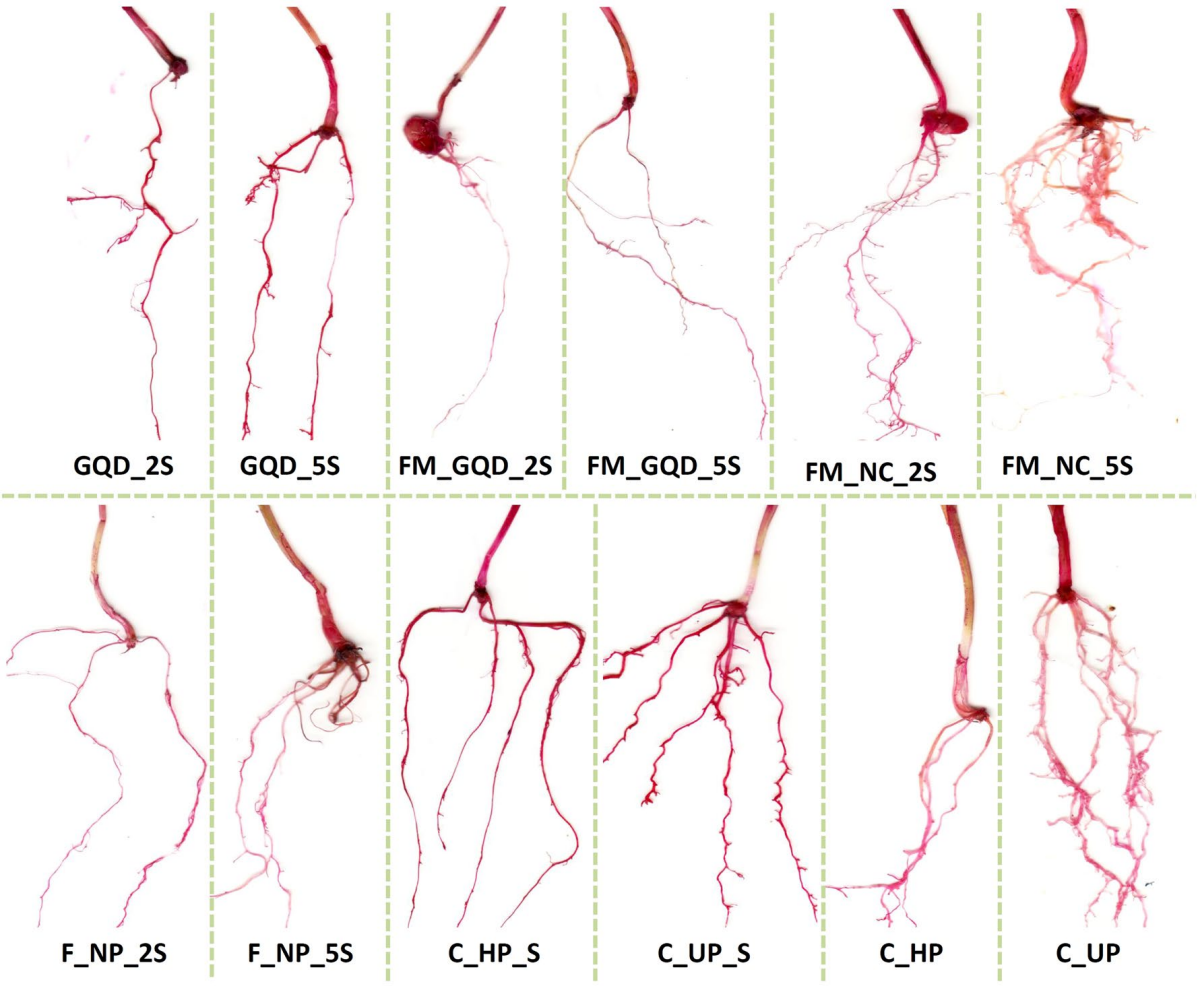
Histochemical detection of lipid peroxidation by using Schiff's reagent in the root of treated wheat seedlings under salinity stress.

**Figure 12:**
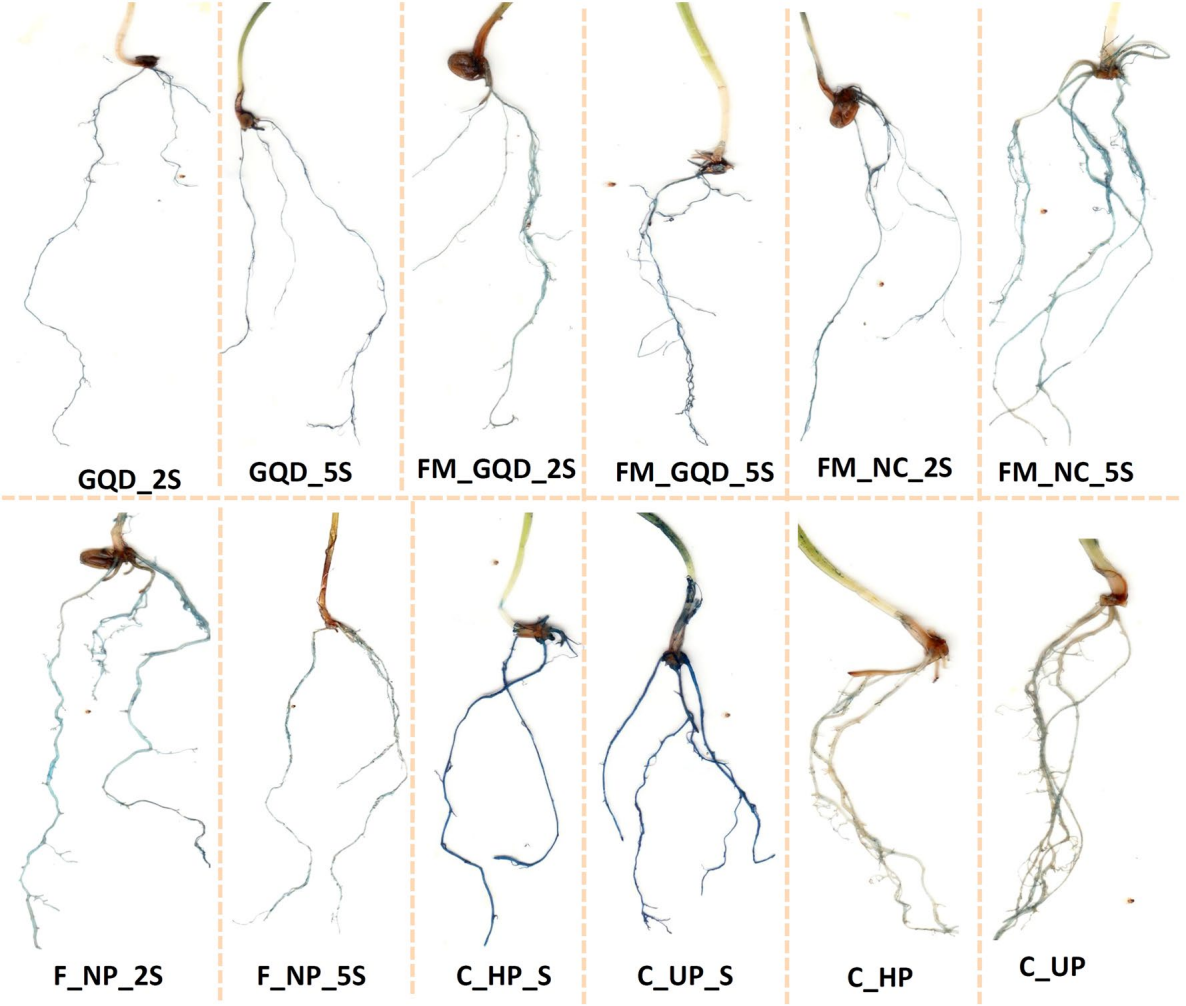
Histochemical detection of plasma membrane integrity by using Evan’s blue stain in the root of treated wheat seedlings under salinity stress.

As discussed previously, being a final product of membrane lipid peroxidation, MDA is used as a bio-indicator to asses stress-induced cell death. Accumulation of MDA in a cell can affect membrane fluidity, thereby limiting the capacity of ionic transport and can cause protein degradation which ultimately leads to cell death^95^. Detection of lipid peroxidation and plasma membrane integrity was done through histochemical staining with Schiff's reagent and Evans blue solution respectively. The actions were noticed exclusively at the root apex and tip. The histochemical staining patterns of lipid peroxidation and loss of membrane integrity were quite similar (Figs. 11 and 12). The use of Evans blue dye has been reported widely as a bio-marker of loss of plasma membrane integrity and cell death. This staining procedure indicated that a decrease in root cell viability under salt stress occurred throughout root cells. The roots of both control and treated wheat seedlings under salinity stress stained darker than the seedling grown in normal physiological conditions (non-saline). Similar results were observed when the roots of the stressed wheat seedlings were treated with Schiff’s reagent.

However, under salt stress, roots of the nanocomposites treated seedlings marked lower Evans blue and Schiff's staining than control saline group which is an indication of less oxidative damage, strong plasma membrane integrity and less lipid peroxidation in treated seedling even under stress condition. These results suggest that the peroxidation of lipids and loss of membrane integrity were caused due to salt stress and nanocomposite treatments to some extent nullifying the generated stress. Ahanger and Agarwal^96^ reported that NaCl-induced salinity stress caused an increase in lipid peroxidation in wheat. Another report suggested that the application of nanoparticles enhanced salt tolerance by lowering the MDA accumulation and through appropriate osmoregulation^97^.

### Assessment of antioxidant enzyme activity

**Figure 13:**
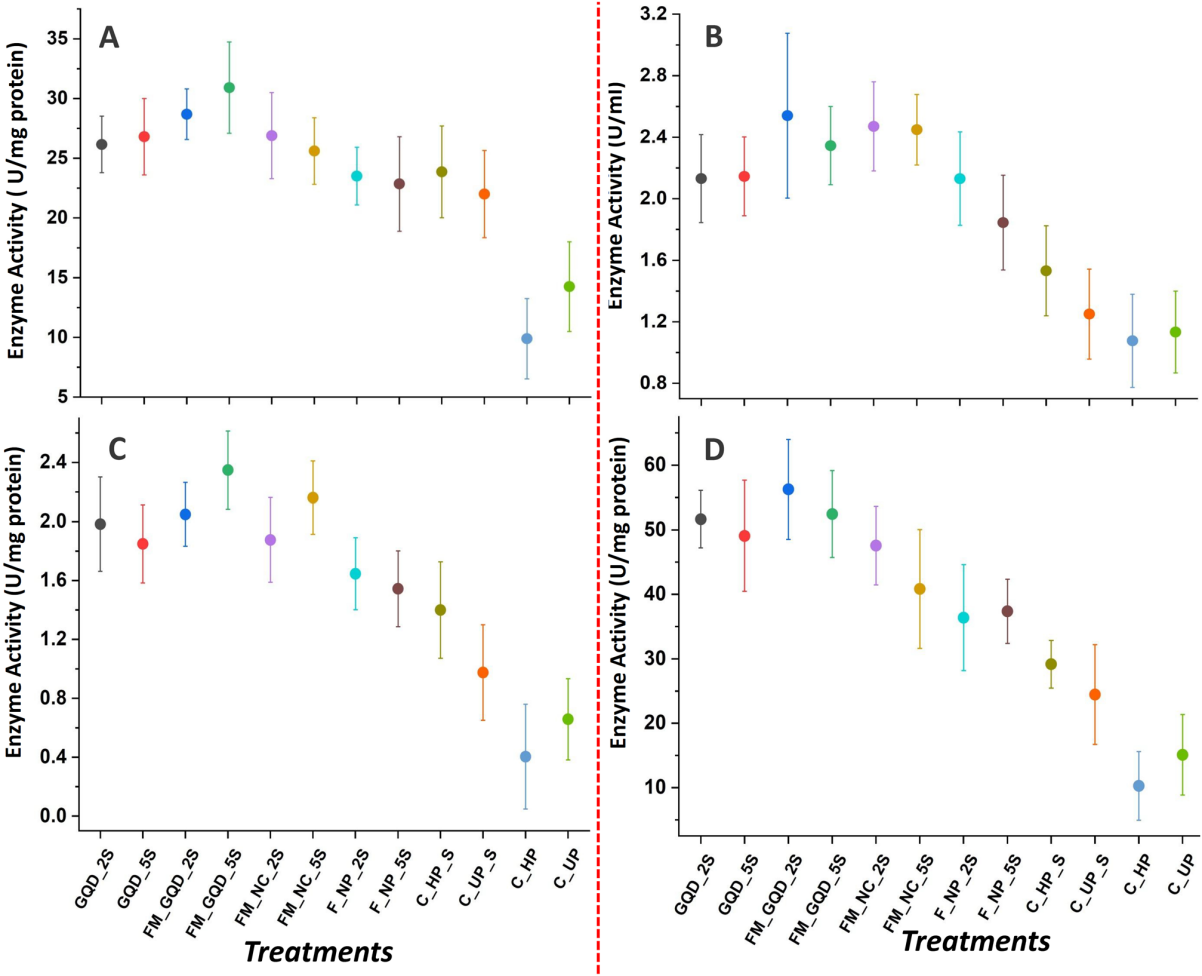
Interval plot represents the effect of applied nanomaterials on various antioxidant enzymes (**A** Catalase, **B** Peroxidase, **C** NADPH Oxidase, **D** Glutathione reductase).

Accumulation of ROS and generation of high osmotic stress in plants is obvious due to excessive salt accumulation in the root surroundings^98^. On accumulation, these ROS enhanced oxidation and degradation of protein and alteration in DNA sequence of plants^99^. By activating a complex array of both non-enzymatic (Such as reduced glutathione, ascorbate, phenols) and enzymatic detoxification mechanisms (such as catalase, peroxidase, NADPH oxidase), plants generally nullify the generated stress^100–102^. Several past studies suggested that the antioxidant defense system in plants plays a potential role in the management of oxidative damage during abiotic stress^103^. Our experimental findings indicated that the activity of all the studied antioxidant enzymes was enhanced under salt stress which was quite similar to the trend^104^. Highest catalase (CAT, EC 1.11.1.6) activity was shown by FM_GQD_5S, whereas the least activity was recorded in the control hydro-primed group and except this, no statistical variation was observed in CAT activity among the seedlings treated with different nanomaterials (Fig. 13A).

Catalase is a heme-containing enzyme catalyzing the decomposition of hydrogen peroxide (H_2_O_2_) into water (H_2_O) and oxygen (O_2_). During photorespiration and β-oxidation of fatty acids, a large quantity of H_2_O_2_ was found to be generated in peroxisome which is generally scavenged by CAT^105,106^. Hydrogen peroxide (H_2_O_2_) is also eliminated by peroxidases (POD, EC 1.11.1.7), catalyzing reduction of H_2_O_2_ using electrons from various reductants^107,108^. From the experimental data it was evident that FM_GQD_2S, FM_NC_2S, and FM_NC_5S showed the same kind of POD activity, and its surpassed the control by 123.98, 117.81, and 115.16% respectively. In the control unprimed group, POD activity did not increase after salinity imposition indicating low defensive activity of the control seedlings (Fig. 13B). Wheat seedlings treated with iron-manganese nanocomposites doped GQD at 500 µg/mL (FM_GQD_5S) recorded the highest for NADPH oxidase (NOX, EC 1.6.3.1) activity, whereas the lower dose of the same treatment i.e. FM_GQD_2S recorded highest for glutathione reductase (GR, EC 1.8.1.7) activity and this particular treatment showed an improvement of 257.11 and 272.58% over control for NOX and GR respectively (Fig. 13C,D). Enhancement in the activity of antioxidant enzymes signified an adaptive mechanism associated with increased plant tolerance to abiotic stress^109^.

### Understanding interactions between different treatments and variables through correlation analysis, PCA, and heatmap-based clustering approach

**Figure 14:**
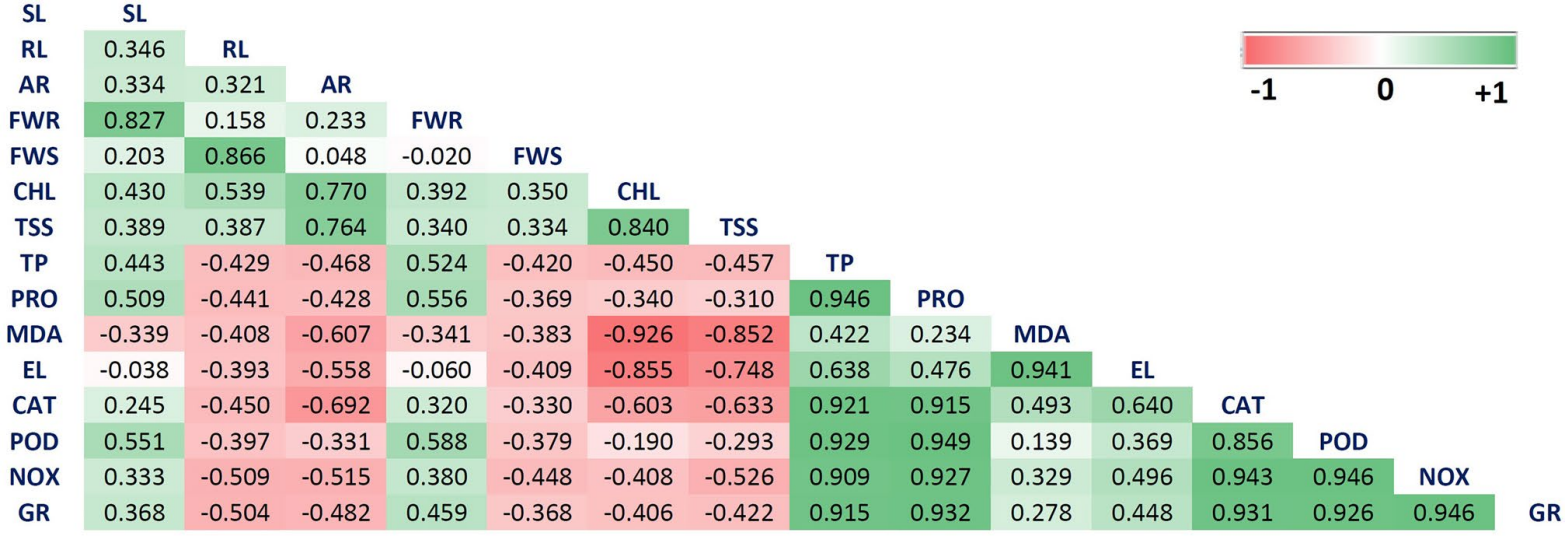
Pearson correlation (at 0.05 level) representing interrelationship among the morphological, biochemical, antioxidant enzymes and stress related parameters (*SL* shoot length, *RL* root length, *AR* number of adventitious root, *FWR* fresh weight of root, *FWS* fresh weight of shoot, *CHL* chlorophyll content, *TSS* total soluble sugar content, *TP* total phenol content, *PRO* proline content, *MDA* malondialdehyde content, *EL* electrolyte leakage percentage, *CAT* catalase activity, *POD* peroxidase activity, *NOX* NADPH Oxidase activity, *GR* glutathione reductase activity).

**Figure 15:**
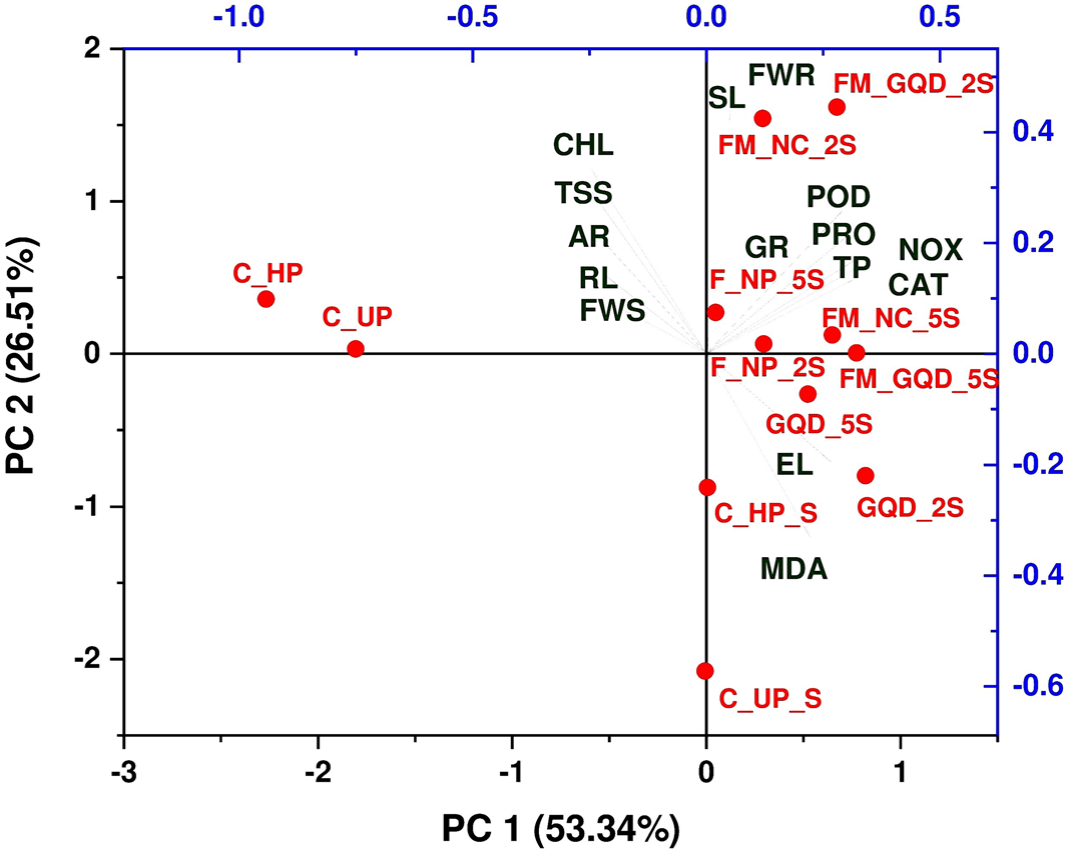
Ordination diagram of principal component analysis (PCA), showing interaction between different treatment solution and variables under study (*SL* shoot length, *RL* root length, *AR* number of adventitious root, *FWR* fresh weight of root, *FWS* fresh weight of shoot, *CHL* chlorophyll content, *TSS* total soluble sugar content, *TP* total phenol content, *PRO* proline content, *MDA* malondialdehyde content, *EL* electrolyte leakage percentage, *CAT* catalase activity, *POD* peroxidase activity, *NOX* NADPH Oxidase activity, *GR* glutathione reductase activity).

Heat map and principal component analysis (PCA) study were used to summarize the actual effect of applied nano-treatments on morpho-physiochemical development of wheat under salt stress, to avail maximum quantity of data variability, and to illustrate an interaction between different treatment solutions and variables. Whereas correlation analysis was carried out to quantify the strength of the relationship between two variables (Fig. 14). The first two principal components i.e. PC1(53.34%) and PC2 (26.51%) were used to make biplots and these two components accounted for a total of 79.85% overall data variability (Fig. 15). From the factor loading value, it was evident that the upper left plot mainly represents the non-saline control group i.e., C_UP and C_HP (PC1 loadings are negative but PC2 loadings are positive). While after salt stress imposition this control group is present alongside on the ‘y-axis’ where the axis value is zero in PC1 and PC2 value is negative. Upper right plot contain FM_GQD_2S, FM_NC_2S, F_NP_5S, FM_NC_5S, and F_NP_2S (both PC1 and PC2 positive). From the PCA of different treatments, it can be concluded that iron-manganese doped graphene quantum dots (FM_GQD_2S) was found to be the most promising treatment solution for wheat salinity stress mitigation as experienced by overall morphological, biochemical, antioxidant enzymes, and histochemical evidence. Also, it can be stated that a lower dosage of treatment solution (here 200 µg/mL) had a more pronounced effect in comparison with a high dose (here 500 µg/mL) which supports the previous findings^110^. GQD when applied in normal form (i.e., without forming composite) did not show marked influences on morpho-physiochemical responses of wheat.

PCA of different variables showed four distinct clusters. First cluster grouped chlorophyll and total soluble sugar content, fresh weight of shoots, and length of roots along with the number of adventitious roots. From the correlation study, it was observed that there was a significant positive correlation (*p* ≤ 0.05) between the fresh weight of shoots and length of roots and among chlorophyll content, total soluble sugar content, and numbers of adventitious roots. Enhancement in chlorophyll and total soluble sugar in treated seedlings and placement of these attributes together with the fresh weight of shoot in the same cluster might be due to the production of active metabolites like reducing sugar (that helps in energy production) accelerated the accumulation of photo-assimilates which eventually leads to an improvement in biomass (fresh weight of shoot) of the seedling. Another major cluster mainly represents enzymatic antioxidants along with total phenol and osmolytes like proline. All the studied enzymatic antioxidants (such as CAT, POD, GR, NOX) exhibit a strong significant (*p* ≤ 0.05) positive correlation among them and with total phenol and proline. The imposition of salt stress and enhancement in active metabolism leads to the production of ROS which ultimately activates the antioxidant enzymes as a part of the defense system. Several past studies suggested that phenolic compounds functioned as a natural antioxidant in various plants and there is a relationship between antioxidant activity and the phenolic content of plants^111,112^.

**Figure 16:**
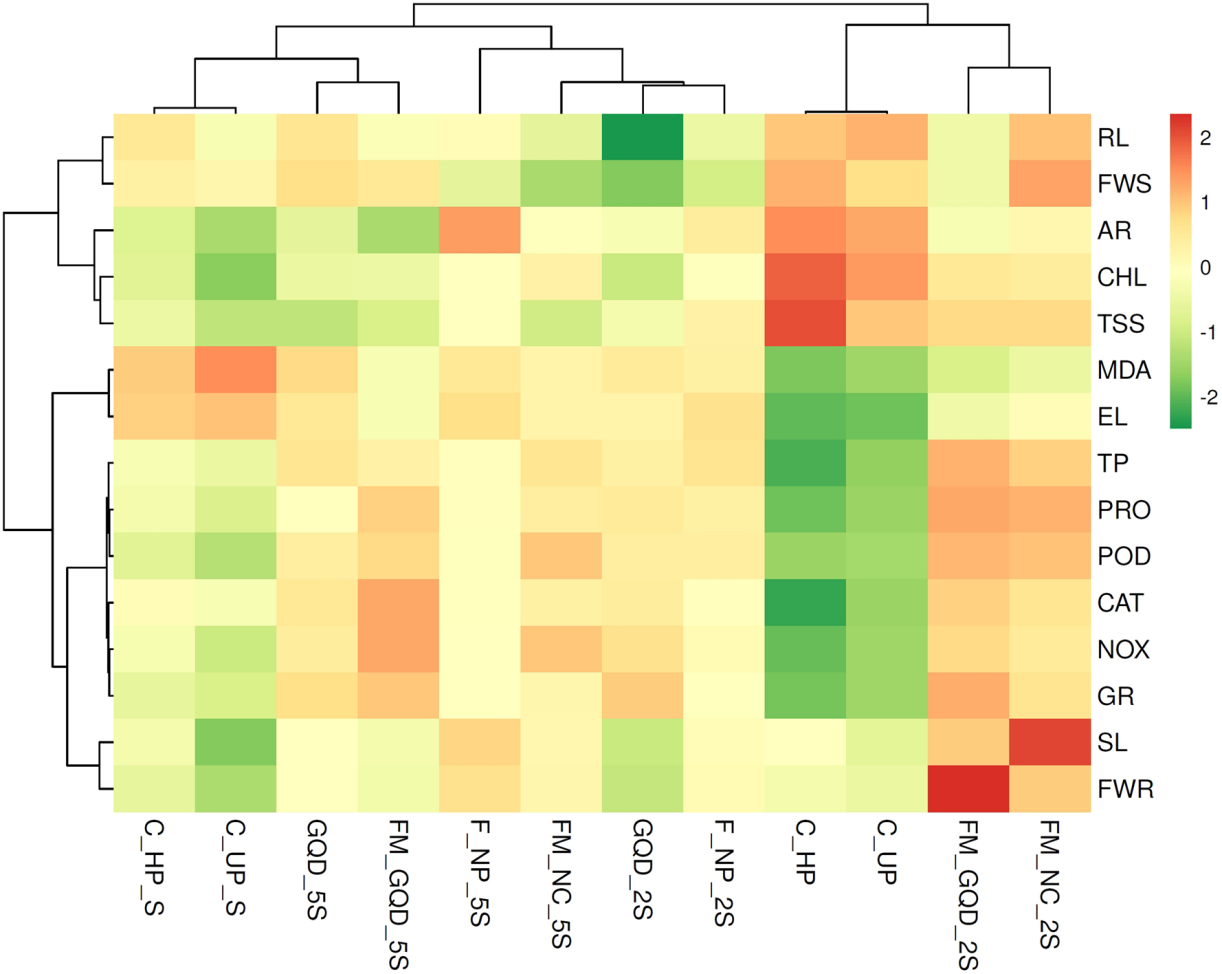
Heatmap analysis of different treatment solution and variables under study (*SL* shoot length, *RL* root length, *AR* number of adventitious root, *FWR* fresh weight of root, *FWS* fresh weight of shoot, *CHL* chlorophyll content, *TSS* total soluble sugar content, *TP* total phenol content, *PRO* proline content, *MDA* malondialdehyde content, *EL* electrolyte leakage percentage, *CAT* catalase activity, *POD* peroxidase activity, *NOX* NADPH Oxidase activity, *GR* glutathione reductase activity).

The presence of MDA and electrolyte leakage (EL) in close proximity as observed through PCA was further supported by correlation analysis which indicates a significant positive correlation between the two variables. Both MDA content and electrolyte leakage increased in response to salt stress and imposed negative effects on plant growth and development. Furthermore, these two variables are negatively correlated with chlorophyll and total soluble sugar. Heatmap is another data visualizing technique used in this study, in which variables and treatment were arranged in rows and columns, respectively (Fig. 16). Hierarchical clustering was shaped based on the closeness of the relationship between variables and treatments. Clusters observed in the heatmap were in agreement with the PCA clustering. The hierarchical clustering in the heatmap indicates that FM_GQD_2S and FM_NC_2S are strongly influencing the mitigation of salinity stress of wheat as these treatments showed more pronounced effects than the control and hydroprimed seedling in normal growth condition (i.e. without salinity stress).

### Probable mechanism of action

Under the current scenario of growing human population and climate change, the use of fertilizers is tremendously increasing as nowadays agriculture primarily relies on chemical input. However, the excessive application of chemical fertilizers has resulted in the discharge of potentially hazardous substances into the environment. With this connection, nanofertilizers have been projected as a sound remedy for tackling the said problem^113,114^. Use of nanoparticles as nanofertilizers has been reported to enhance tolerance against various abiotic stresses including salinity^113^. El-Ashry et al.^115^ on the application of nitrogen-based nanofertilizer under salinity stress observed an improvement in wheat plant growth, grain dry matter, and nutritional quality. Soliman et al.^116^ reported that the salt stress of *Moringa* can be relieved through foliar supplementations of Fe_3_O_4_ and zinc oxide nanoparticles via improving enzyme activity. Applications of carbon-based nanoparticles have also been reported to alleviate unfavorable environmental conditions in plants, especially salinity stress^11^. Previous reports suggested that, in wheat, salinity stress reduces germination, nutrient uptake, relative water content, and photosynthesis, and also induced toxicity and oxidative stress which eventually leads to a reduction in growth and final yield. Salinity stress creates an imbalance between ROS and antioxidants which accelerates oxidative stress in the wheat crop. Furthermore, salt stress enhances ionic toxicity, reduces leaf growth, and enforces early leaf abscission, which decreases the rate of carboxylation and photosynthesis. In addition to that, salt stress also reduces the efficacy of PS-II, intercellular CO_2_, stomatal conductance, and electron transportation; all these cumulatively or individually contribute towards a reduction in photosynthesis^117^.

**Figure 17:**
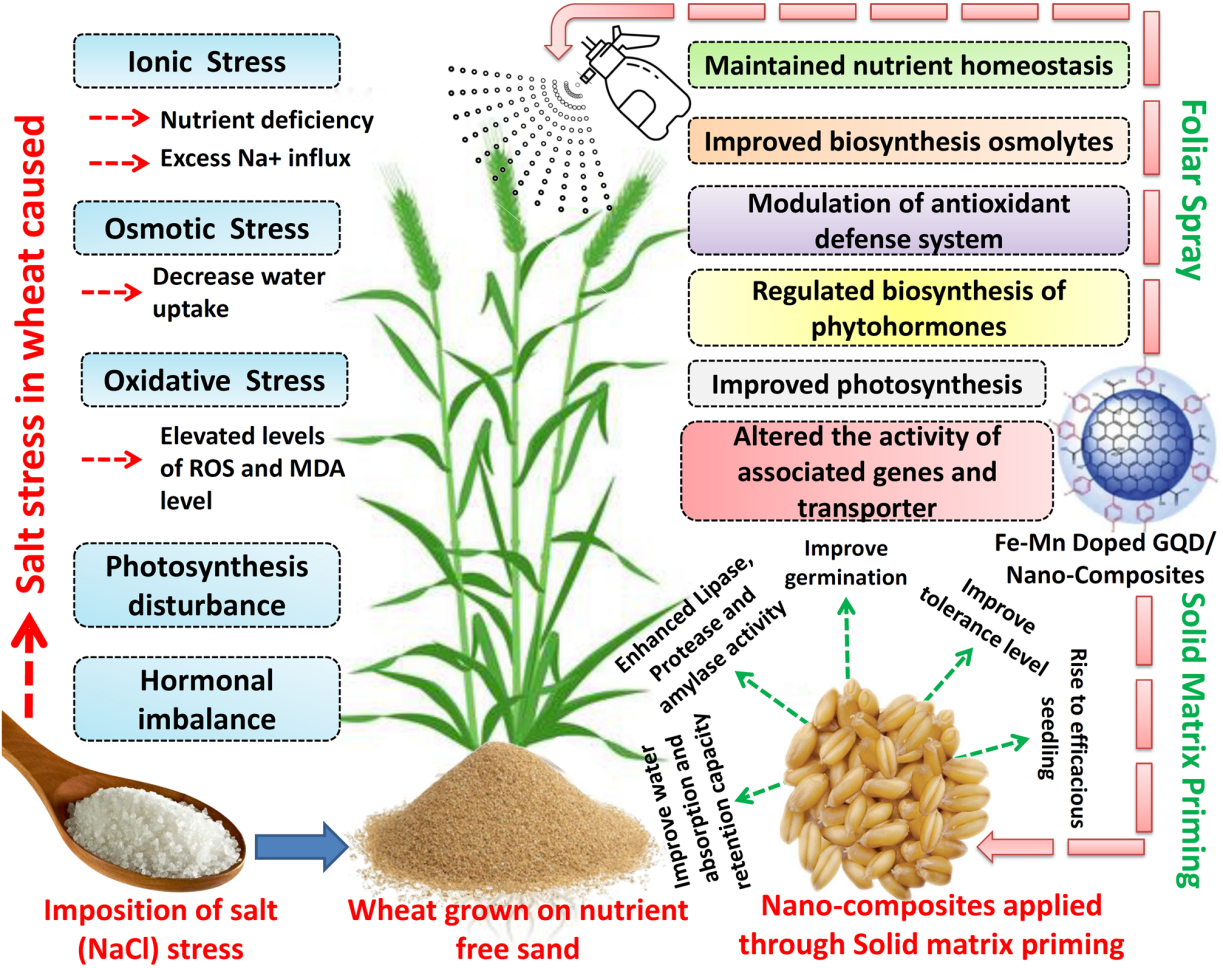
Probable mechanism of action of the applied nanomaterials on wheat seedling growth and salinity stress mitigation.

To counteract this salinity-induced stress, micronutrient like iron and manganese nanocomposites doped graphene quantum dots along with their individual form (i.e. GQD, Fe–Mn nanocomposites, iron nanoparticles) were applied through solid matrix priming. Nano-priming enhanced lipases, amylases, and proteases enzyme activities that degrade macromolecules to facilitate the growth and development of embryos. Increased rate of germination in nano-primed seeds indicated early availability of nutrient-rich solutes nullified the generated stress at the germination stage and eventually results efficacious seedling formation^118^. Application of nanoparticles also improves water absorption and retention capacity of seeds^63^. Previous study reported that carbon nanotubes treatments in tomato seeds enhanced seed moisture content by 199% in comparison to untreated seeds^81^. This result suggested that nanoparticles improve uptake and retention of water may be by creating water permeation channels into the seed coats^119^. Though the mechanism is not fully understood but it is assumed that nanoparticles regulate the aquaporins in the seed coats^63^. Martínez-Ballesta et al.^120^ reported that application of carbon nanotubes improve aquaporin transduction in broccoli, which leads to enhanced water uptake and thereby mitigate salt stress. Nanocomposites doped GQD applied through foliar spray cross cuticular barrier following the lipophilic or hydrophilic pathway^121^. In hydrophilic pathway, applied nanomaterials were dispersed through polar aqueous pores of cuticle or stomata, whereas lipophilic pathway involves diffusion of the nano molecules through cuticular waxes^122,123^. After entering the leaf apoplast these particles may undergo long distance transport through vascular system. Past reports suggested that photosynthate, sugars and other macromolecules located in the leaf can be transported downward through the phloem to the shoot and root^124^. This down-flow and foliar uptake of nanoparticles can be supported by the experimental evidence of Wang et al.^125^ in which four kinds of metal oxide nanoparticles applied on leaves can successfully penetrate the leaves and can reach the shoots and roots. On reaching the plant system, nano-materials interact with plants at cellular and sub-cellular levels and initiate a series of activities in morphological, physiological, biochemical, and molecular states^126^. Past evidence suggested that different nanoparticles applied at concentrations below certain limits can promote plant growth and development under saline condition^62,63,127^ by various known mechanisms (Fig. 17). Applied nanoparticles inside the plant system maintain nutrient homeostasis and provide energy during environmental stress^128^. Regulated biosynthesis of osmolytes and osmoprotectant is another mechanism by which nanoparticles provide tolerance during salinity stress^62^. Increased accumulation of osmolytes like proline as evident from experimental data may facilitate in the maintenance of ironic homeostasis in the plant body and thereby reduces the osmotic shock generated by salt (NaCl) stress due to ion toxicity (Na^+^ and Cl^−^)^81^. Glycophytes like rice, and wheat are extremely susceptible to salt stress-induced oxidative injuries, and in response to that different enzymes (such as catalase, superoxide dismutase) get activated to mitigate the generated stress. On application of nanocomposites doped GQD, the activity of several antioxidant enzymes increased (as experienced from experimental results) which provides a buffered redox system inside the plant body^129^.

Indeed a close association between antioxidants (enzymatic and non-enzymatic) and salinity tolerance has been observed in wheat species^130^. Nanoparticles also regulate the biosynthesis of phytohormones, maintain plant water balance and improve water use efficiency^62,81,131^. Moreover, carbon-based nano-materials have been found to regulate gene expression under salinity stress. Application of multi-walled carbon nanotubes on salt-stressed rapeseed seedlings changes the expression of the salt overly sensitive 1 (SOS1) gene, and also altered the activity Na^+^/H^+^ exchanger 1 (NHX1) and K^+^ transporter 1 (KT1) transcripts^132^. This application encourages intensification of nitrate reductase-dependent nitric oxide (NO) biosynthesis, and restoration of ion and redox balance as evidenced by the decrease in ROS production and decrease in Na^+^/K^+^ ratio^132^. All of these phenomena play an ameliorating role in adaptation during salinity stress.

## Conclusion

In summary, this study reported a quick and simple method of GQD and its nanocomposite synthesis from easily available natural sources. This study also demonstrated the use of GQD and its composite form in the alleviation of salt stress and enhancement of wheat seed germination and seedling growth. Our observation depicted that iron-manganese nanocomposites and iron-manganese nanocomposites doped GQD substantially improved different phenotypic characteristics of the studied plants. Besides lowering the generation of stress under saline condition, these treatments also exhibited a greater impact on different biochemical, enzymatic, and non-enzymatic antioxidant attributes. From the overall observation, it might be concluded that lower dosage (200 µg/mL) of the effective treatments (FM_NC and FM_GQD) were more pronounced than the higher ones. Furthermore, these nanocomposites might serve as an ideal substitution for the traditional chemical fertilizer and will be helpful in the nutritional fortification of plants. However, some points like the incorporation and translocation, and accumulation of these nanocomposites inside the plant systems should be investigated. Our future aspect includes the determination of slow and controlled release (of incorporated nutrient) efficiency of the synthesized nanocomposites, large-scale field trials in different locations, and implementation in the field as well as release through industries for farmer's use.

## Data Availability

All the associated with this work are presented here and further will be made available on reasonable request. Crystallographic data has been deposited in ICDD database and can be accessed by logging into ICCD database (https://www.icdd.com/submission/contributors/submission_display_cur.php?subid=71 Submission ID: MDH1671109280).
